# Trophic dynamics and metabolic pathways in host parasite interactions revealed by nitrogen isotope analysis of amino acids in multiple tissues

**DOI:** 10.1038/s41598-025-19052-0

**Published:** 2025-09-18

**Authors:** Shaista Khaliq, Milen Nachev, Philip M. Riekenberg, Maik A. Jochmann, Maryam Vosough, Frederik Franke, Jörn P. Scharsack, Joachim Kurtz, Bernd Sures, Marcel T. J. van der Meer, Torsten C. Schmidt

**Affiliations:** 1 Instrumental Analytical Chemistry, University of Duisburg-Essen, Universitätsstr. 5, 45141 Essen, Germany; 2 Aquatic Ecology, University of Duisburg-Essen, Universitätsstr. 5, 45141 Essen, Germany; 3 Centre for Water and Environmental Research, University of Duisburg-Essen, Universitätsstr. 5, 45141 Essen, Germany; 4 Marine Microbiology and Biogeochemistry Department, NIOZ Royal Netherlands Institute for Sea Research, 1790 AB, Den Burg, The Netherlands; 5 Center for Applied Isotope Studies, University of Georgia, Athens, GA 30602 USA; 6 Research Center One Health Ruhr, Research Alliance Ruhr, University of Duisburg-Essen, 45141 Essen, Germany; 7 Institute for Evolution & Biodiversity, University of Münster, Hüfferstr. 1, 48149 Münster, Germany; 8 Present Address: Thünen Institute of Fisheries Ecology, Herwigstr. 31, 27572 Bremerhaven, Germany; 9 Present Address: Bavarian State Institute of Forestry, Hans-Carl-von-Carlowitz-Platz 1, 85354 Freising, Germany

**Keywords:** Host-parasite interactions, Food webs, Metabolic pathways, Stable isotope, GC-C-IRMS, Cestode, Ecology, Ecology, Limnology

## Abstract

Trophic interactions are crucial for understanding food web complexity and energy flow in ecosystems. Including parasites in these analyses can alter dynamics and challenge ecological assumptions. Compound-specific isotope analysis offers greater precision than bulk stable isotope analysis by analyzing individual amino acids, providing deeper insights into nutrient exchange and metabolism in host-parasite systems. In this study, we conducted a 120-day controlled feeding experiment to investigate parasite metabolic pathways, host-parasite trophic dynamics, and trophic fractionation between infected and uninfected sticklebacks. Nitrogen isotope composition (δ^15^N) was measured in host *Gasterosteus aculeatus* liver and muscle tissues, and its ‘cestode’ parasite (*Schistocephalus solidus*). Parasite serine δ^15^N values were higher by 4.4 ± 2.4‰ compared to host liver, indicating a strong metabolic link. Lower proline concentrations and increased alanine δ^15^N values (5‰) indicate support of parasite growth through conversion to hydroxyproline and increased gluconeogenesis. The trophic position difference between parasite and host tissues was < 0.5, suggesting direct assimilation of host-derived products. Infected host tissues exhibited ~ 5‰ increase in glycine δ^15^N over time compared to control tissues, likely reflecting the host’s increased metabolic demand for immune support during infection. This study highlights the complex metabolic interactions in host-parasite systems.

**Supplementary Information:**

The online version contains supplementary material available at 10.1038/s41598-025-19052-0.

## Introduction

Food webs serve as a foundational ecological concept, providing a framework for understanding the complex trophic interactions within a community of organisms^1^. Structures within food webs are crucial for interpreting energy flow through ecosystems and predicting the consequences of disturbances^2^. Traditionally, food web studies have predominantly focused on predator–prey and herbivore-primary producer relationships. Studies often inadvertently overlook the significant role of parasitic interactions, for example, direct energy transfer, and indirect feeding of other predators^3–5^. However, parasites can induce substantial variations in trophic structure and dynamics, challenging fundamental ecological assumptions^6–9^. Because many parasites depend on host-derived nutrients and possess specialized metabolic strategies for nutrient assimilation, they interact with host biochemistry differently from conventional consumers^10,11^. These interactions can shift host energy use, alter apparent trophic positions, and introduce unexpected nutrient flow pathways, making parasites integral components of food webs^7^.

Stable carbon and nitrogen isotope analyses (δ^13^C and δ^15^N) have emerged as valuable tools for investigating food web dynamics. They reveal distinct trophic niches and transfer pathways within ecosystems^12,13^. Bulk stable isotope analysis has been widely used to gain insights into energy and nutrient exchanges within host-parasite systems^14^. Bulk stable isotope analysis assesses the target element across all compounds in a sample as a weighted average, which simplifies analysis^15^ but assumes a standardized trophic enrichment factor, a stepwise increase (~ 3.4‰) in δ^15^N values per trophic level^16^. This assumption aids in broad trophic estimation, but discrepancies arise in host-parasite systems where δ^15^N enrichment may vary substantially, requiring specific enrichment factors for accuracy^5,17,18^. Despite the ecological roles parasites play, these trophic complexities are still understudied^8,18^. Studies like Born-Torrijos et al.^9^ outline potential metabolic and behavioral processes that can lead to changes in stable isotopic composition of host tissues and identifies potential mechanisms driving these shifts in host-parasite systems.

Compound-specific stable isotope analysis (CSIA) has emerged as a complementary approach to address the limitations of Bulk stable isotope analysis^19^. Compound-specific stable isotope analysis offers valuable information on a variety of variables by isolating specific compounds, such as amino acids, from complex mixtures^20^. This biochemical building-block approach of CSIA allows for more precise tracing of molecular exchange within the food web^20,21^.

The compound-specific analysis of stable isotopes of nitrogen from amino acids (δ^15^N-AA) can be divided into three groups. (i) Source amino acids (SAA), which retain their δ^15^N base level, and (ii) trophic amino acids (TAA) which show a significant enrichment of 4 to 8‰ by transamination.^22^ (iii) Metabolic amino acids (MAA) undergo significant isotopic changes due to internal metabolic processes that are not associated with transamination^23^.

For the estimation of trophic position, many studies have relied on the difference between glutamic acid (Glu) and phenylalanine (Phe) as recognized TAA and SAA^22,24,25^. The difference between these amino acids can effectively indicate the trophic position of an organism (i.e., TP_Glu/Phe_), even in systems with variable nitrogen isotope baselines^15,26^. However, alternative combinations or multiple amino acid approaches have also been applied^27–29^, that apparently reduce variability compared to individual amino acid or bulk-SIA measurements, thus potentially improving the accuracy of trophic position estimations. Despite the potential of CSIA, the application of δ^15^N-AA analysis in parasite studies is still limited, emphasizing the need for further exploration and understanding^14^.

Notably, most ecological studies using stable isotopes analyze only whole-body or muscle tissue^30,31^. Muscle tissue, typically favored due to its accessibility, exhibits relatively low turnover and incorporation rates, rendering it indicative of longer-term dietary intake and less sensitive to minor or seasonal dietary changes in isotope values^32^. Conversely, the liver exhibits an instant response to dietary fluctuations owing to continuous protein turnover and regulatory activities, rendering it a valuable indicator of short-term dietary changes^32–34^. Moreover, the liver’s pivotal role in amino acid metabolism and biosynthesis underscores its significance in unraveling dietary dynamics^35^. However, the increased isotopic shifts observed in liver samples compared to muscle samples may be attributed to their lipid content, as lipid extraction can significantly alter δ^15^N values^32,36^. Despite these challenges, lipid extraction is a necessary process, and once completed, the liver tissue can still provide valuable insights into dietary dynamics of consumer protein metabolism^37–39^. In a study of host-parasite interactions, such as those involving cestodes as parasites, liver δ^15^N turnover rates have been found to be the highest, followed by muscle tissue^37^. In this study, perch without parasites (from rearing) and wild-caught perch with spade tapeworm (Triaenophorus nodulosus, lives in the liver) were examined. In general, the isotope turnover rates for δ^15^N and δ^13^C were fastest in the liver, followed by blood and muscle. Infected hosts demonstrate liver δ^15^N turnover rates that are three to five times faster than those of uninfected subjects, indicating a heightened level of protein metabolism in the liver compared to muscle tissue^37^. The liver’s δ^15^N half-life is approximately 16 days, while muscle tissue, by comparison, has a δ^15^N half-life of about 56 days, illustrating a significantly slower turnover rate^37^.

In our current investigation, we delve into the interaction between the host (three-spined stickleback) (*Gasterosteus aculeatus*) and the cestode parasite (*Schistocephalus solidus*), using amino acid nitrogen isotope analysis. This well-established model system is critical for studying the ecology and evolution of host-parasite interactions^40^. In our previous work, we used the same model to conduct amino acid carbon isotope analysis, examining isotope fractionation across multiple host tissues^41^ and tracing nutrient flow within the host-parasite relationship^10^. *Gasterosteus aculeatus* is the highly specific, second intermediate host of the cestode *S. solidus*, in which the parasite can grow more then 10,000 fold in the body cavity^42^. In extreme cases and multiple infections, the mass of the parasites can reach 50% of the host mass^43^. Significant growth of *S. solidus* in its stickleback host benefits its reproductive output, since larger worms produce higher numbers of eggs in the final host, a fish eating bird^44^. The eggs are shed with the birds feaces and free swimming larvae hatch, which infect cyclopoid copepods as first host^45^. When a three-spined stickleback subsequently eats an infected copepod, the procercoid penetrates the fish’s intestinal wall and develops into a plerocercoid larva within the abdominal cavity^44,46^. The parasite remains free-floating, bathed in host fluids, and absorbs nutrients via a syncytial, microtrich-covered tegument without embedding in host tissues^47,48^. The life cycle is completed when the infected stickleback is consumed by an avian piscivore, where the plerocercoid matures and reproduces in the bird’s intestine^44^. With the present study, we used nitrogen isotope analysis of amino acids to uncover unique trophic dynamics and metabolic pathways in the interaction of *S. solidus* with its stickleback host. It is a novel application of a long-term feeding study applying δ^15^N amino acid analysis to multiple tissue types. Our study examines both liver and muscle tissues to provide a broader understanding of host-parasite interactions. Through the application of δ^15^N CSIA of amino acid, we aim to investigate (1) trophic fractionation (Δ^15^N) between consumer tissues and diet in both control and infected systems, (2) Δ^15^N fractionation patterns between host tissues and parasite across infection stages, (3) the development of trophic dynamics throughout infection, and (4) differences in Δ^15^N between infected and non-infected host tissues.

## Results

### Δδ^15^N fractionation between consumer tissues and diet

**Fig. 1:**
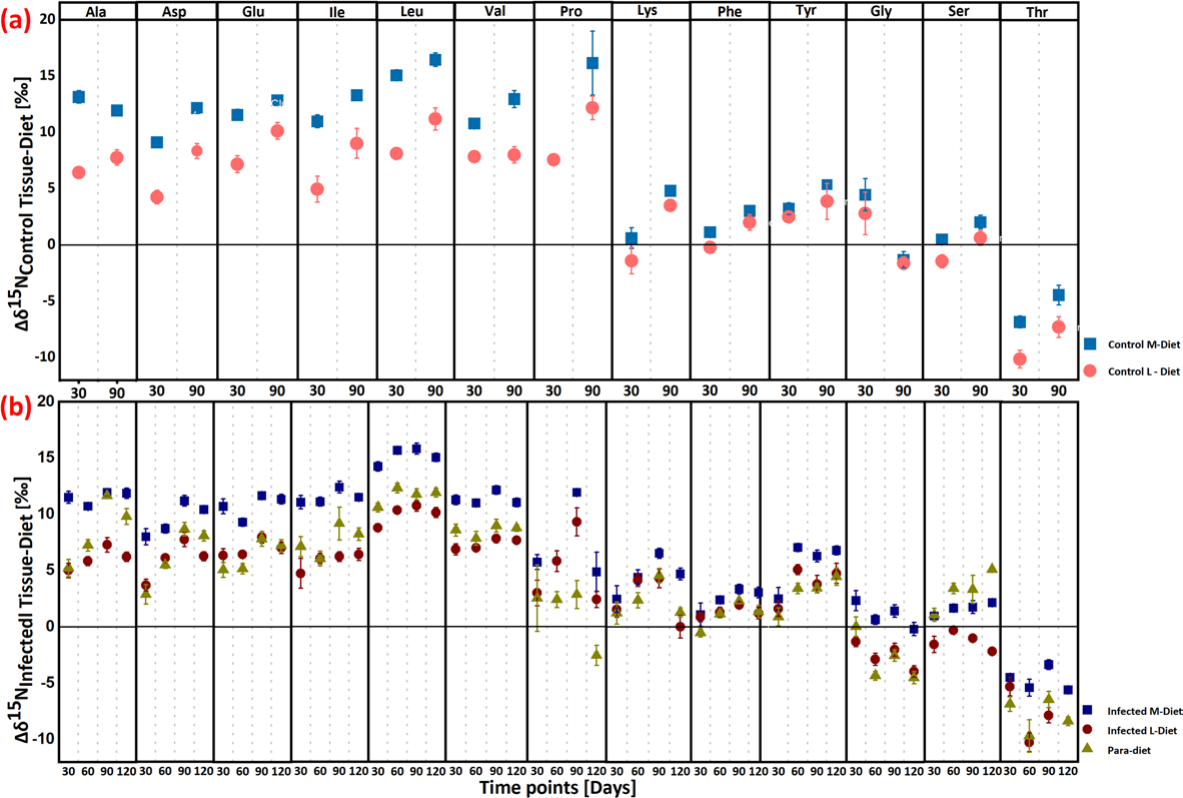
Mean trophic fractionation (Δδ^15^N ± standard error (SE) in‰) of individual amino acids (**a**) between control tissues (n = 3) and diet (mosquito larve; n = 1) at 30 and 90 days; where M = host muscle tissue and L = host liver tissue. (**b**) between infected tissues and parasite (n = 4) and diet (mosquito larve; n = 1) at 30, 60, 90, and 120 days; where Para = parasite. Amino acid abbreviations: Ala = Alanine, Asp = Aspartic acid, Glu = Glutamic acid, Ile = Isoleucine, Leu = Leucine, Val = Valine, Pro = Proline, Lys = Lysine, Phe = Phenylalanine, Tyr = Tyrosine, Gly = Glycine, Ser = Serine, Thr = Threonine.

Trophic fractionation (Δ^15^N) of amino acids was evaluated in both control and infected host tissues relative to their diet (sample of mosquito larvae; n = 1). In control hosts, fractionation between muscle and liver tissues (n = 3) and diet was calculated on sampling days 30 and 90 (Table S5, Fig. 1a). For infected hosts, fractionation between muscle, liver, and parasite tissues (n = 4) and diet was assessed on sampling days 30, 60, 90, and 120 (Table S6, Fig. 1b). Based on Δδ^15^N differences between consumer tissues and diet, three distinct categories of amino acids were identified. TAAs, including Alanine (Ala), Aspartic acid (Asp), Glutamic acid (Glu), Isoleucine (Ile), Leucine (Leu), Valine (Val), and Proline (Pro), exhibited higher fractionation in both control and infected consumer tissues compared to their diet, while SAAs, including Lysine (Lys), Phe, Tyrosine (Tyr), Glycine (Gly), and Serine (Ser), displayed lower isotopic fractionation. An additional, more unusual category, MAAs, includes Threonine (Thr), which showed significant negative fractionation in consumer tissues relative to dietary Thr, with Δδ^15^N values of − 6.9 ± 0.5‰ in control muscle tissue and − 10.2 ± 0.8‰ in control liver tissue after 30 days. For TAAs, the fractionation of control and infected muscle tissue relative to diet (Δδ^15^N_Control & Infected M–Diet_) increased up to approximately 16‰, while control and infected liver tissue and parasite relative to diet (Δδ^15^N_Control & Infected L–Diet_ and Δδ^15^N_Parasite–Diet_) increased to around 12‰. For SAAs, Δδ^15^N values increased from 0‰ to roughly 4–5‰ across both control and infected conditions. Notably, Pro and Ser in parasite tissue deviated from these general amino acid categories: Pro exhibited Δδ^15^N_Parasite–Diet_ below 3‰ at all time points, whereas Ser showed Δδ^15^N_Parasite–Diet_ values exceeding 5‰ after 120 days. Analysis of Variance (ANOVA) simultaneous component analysis (Fig. 3) further supported the categorization of amino acids across the host-parasite system, confirming these distinct patterns in parasite tissue.

Additionally, Fractionation of most amino acids in the infected system increased from 30 to 90 days post-infection relative to the diet, then either decreased or stabilized (Δδ^15^N_Infected Tissue–Diet_ < 0.5‰) between 90 and 120 days post-infection. Exceptions included Gly, where Δδ^15^N_Infected Tissue–Diet_ values continuously decreased over time, and Ser and Tyr, where Δδ^15^N_Parasite–Diet_ values consistently increased. In control tissues, Δδ^15^N_Control Tissue–Diet_ also rose from 30 to 90 days, except for Gly in both liver and muscle tissues and Ala in muscle tissue. Overall, muscle tissue exhibited greater fractionation relative to diet than liver tissue and parasite tissue, with Leu in control muscle showing a pronounced increase in Δδ^15^N, reaching 16.5 ± 0.6‰ after 90 days. The observed higher Δδ^15^N fractionation in muscle relative to diet, compared to liver, suggests a slower turnover rate in muscle tissue. Conversely, liver tissue exhibited more dynamic shifts in Δδ^15^N, indicative of a faster turnover rate. For example, Ala fractionation in infected muscle tissue relative to diet (Δδ^15^N_InfM–Diet_) was less than 0.5‰, while infected liver tissue showed Δδ^15^N_InfL–Diet_ values exceeding 2‰ after 90 days. Finally, most amino acids in parasite tissue showed a similar turnover rate pattern to that of infected liver tissue relative to diet, as evidenced by Δδ^15^N_Para–Diet_ values, highlighting a close metabolic alignment between parasite and liver tissue.

### Δδ^15^N fractionation between host tissues and parasite

**Fig. 2:**
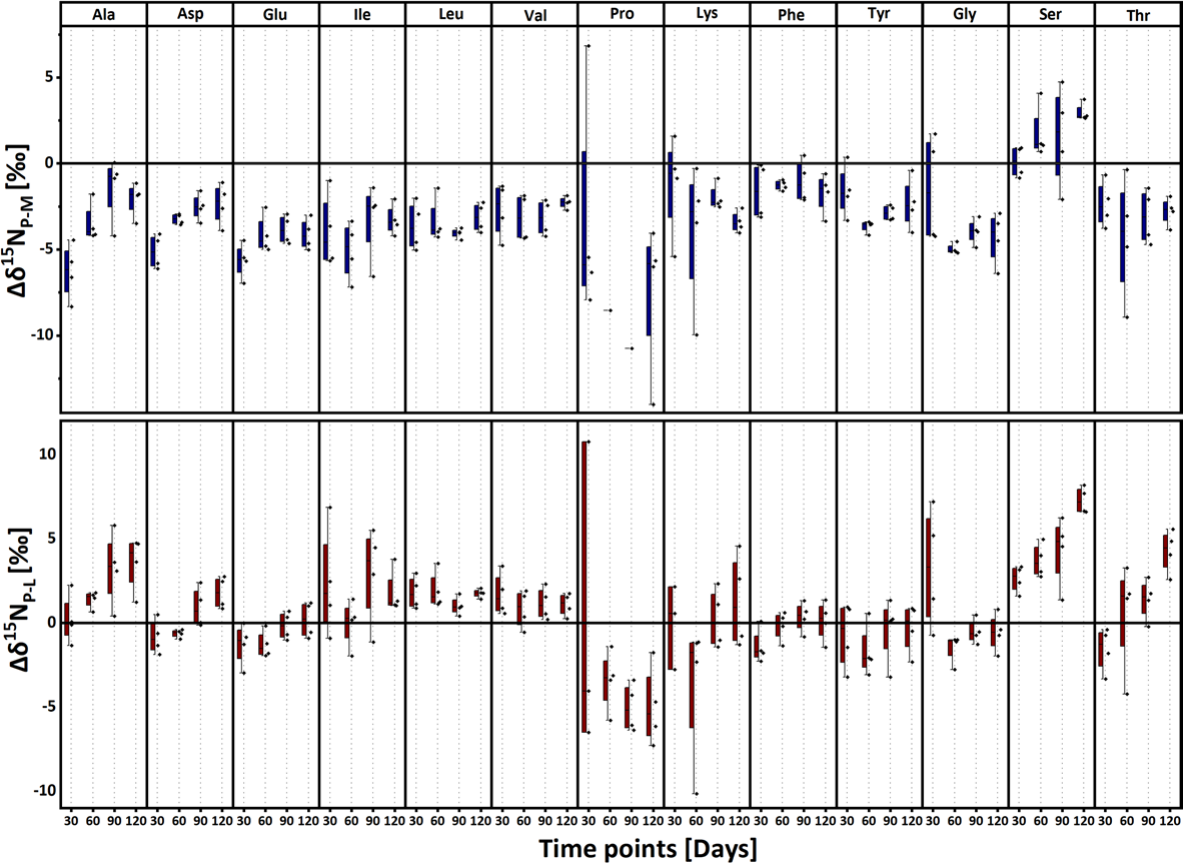
Box plots representing mean trophic fractionation (Δ^15^N in ‰) of individual amino acids at 30, 60, 90, and 120 days. Upper) between parasite (P) and infected host muscle tissue (M). Lower) between parasite (P) and infected host Liver tissue (L). n = 4 for each sample type at each time point.

Paired sample t-tests were used to assess the mean differences in isotope ratios between the parasite and host muscle tissue (Δδ^15^N_P-M_) and between the parasite and host liver tissue (Δδ^15^N_P-L_) relative to zero, with a 95% confidence interval (*p* < 0.05) (Table S3). The Δδ^15^N between parasite and infected host tissues across all time points (30, 60, 90, and 120 days post-infection) were plotted for each amino acid (Fig. 2). Parasite tissues had generally lower δ^15^N values than muscle tissues for most amino acids, except for Ser, which showed an increase of 3.0 ± 0.5‰ in Δδ^15^N_P-M_ at 120 days post-infection. Pro, on the other hand, demonstrated a decrease of 7.4 ± 4.5‰ at 120 days post-infection. When comparing parasite and liver tissues, most TAAs (Ala, Asp, Glu, Ile, Leu, and Val) exhibited higher isotope ratios in the parasite at 120 days post-infection, with enrichments generally under 2‰, except for Ala. In contrast, Pro demonstrated a significant decrease of 5 ± 2.4‰ during the same period. Among the other amino acids, Ser exhibited the most significant increase (7.3 ± 0.8‰), followed by Thr (4.3 ± 1.3‰), suggesting their distinct metabolic roles in the parasite. Additionally, Ala showed a consistent increase throughout the infection, rising by around 4‰ compared to liver and 5‰ compared to muscle tissue from 30 to 120 days post-infection.

### Assessment of the effects of experimental factors by ANOVA simultaneous component analysis

**Fig. 3:**
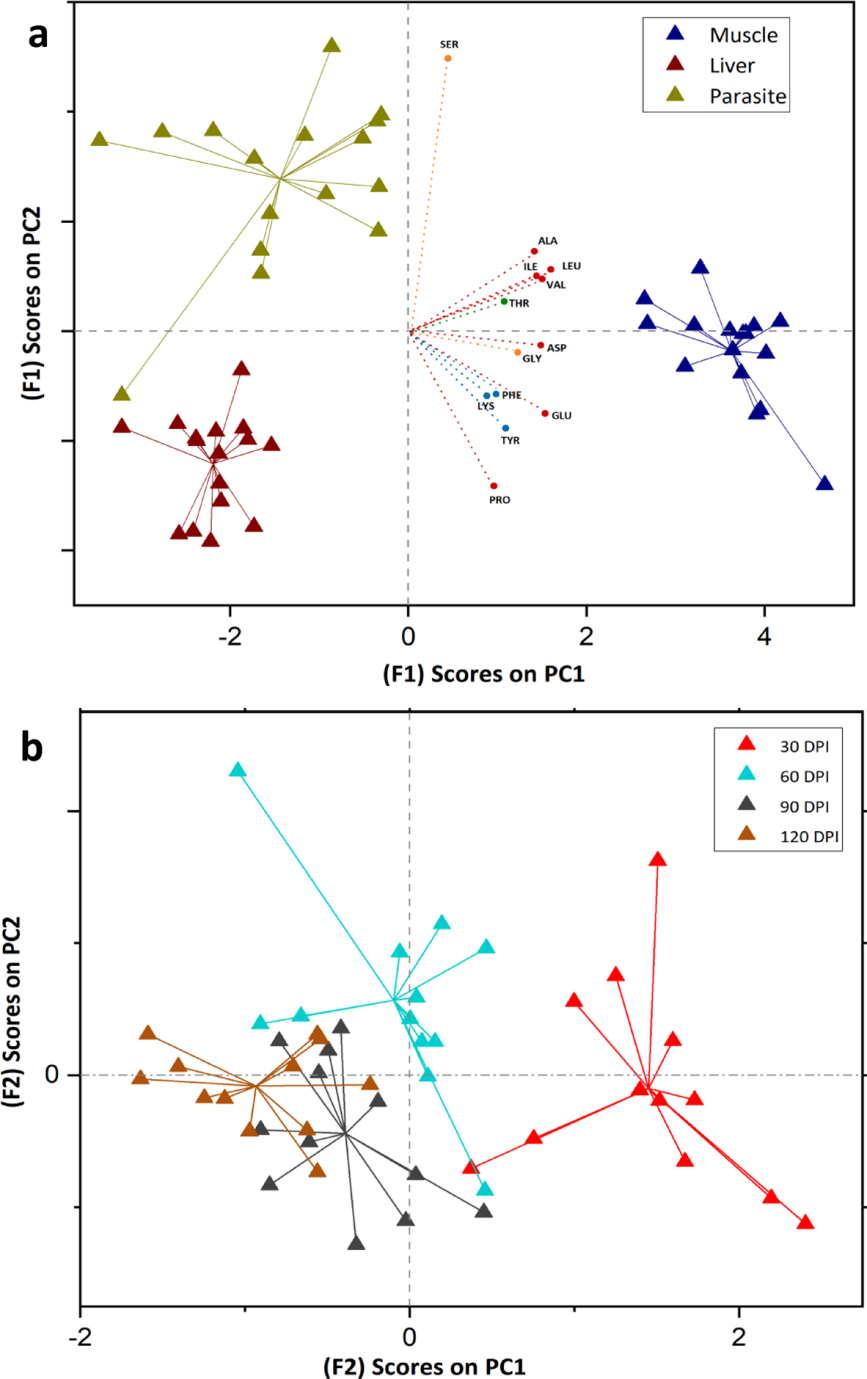
(**a**) ANOVA simultaneous component analysis (ASCA) biplot of sample scores for factor 1 (sample type) shows separation between liver, muscle, and parasite samples along PC2. Amino acids are classified into Source (Phe, Lys, Tyr), Trophic (Ala, Ile, Leu, Val, Asp), and Metabolic (Gly, Ser), with deviations for Pro and Glu. Thr is categorized separately due to its unique diet-consumer interactions. Ser’s association with the parasite suggests its distinct metabolic role, while Pro indicates unique metabolic behavior in host-parasite interactions. (**b**) ANOVA simultaneous component analysis sample (ASCA) score plot for factor 2, reflecting infection times (30, 60, 90, and 120 days).

A first impression of the amount of variation related to the factors sample type and infection time was obtained by partitioning the total data variation into contribution from these factors and their possible interaction. Significant variations were observed in both factors (sample type and infection time), as well as their interaction (*p* < 0.001 in all cases). Sample type (F1) was found to be the dominant source of variation, explaining 61.4% of the variation, followed by infection time (F2) having an effect of 7.9% and an interaction effect of 7.5%. In addition, 23.1% of variation in the data can be attributed to experimental uncertainty (residuals) in the data set. An ANOVA simultaneous component analysis biplot of two components of factor “Tissue type” shown in Fig. 3a, indicates clustering of host tissues and parasite samples along the principal components. Muscle samples show high discrimination along PC1 (83% of F1 variation), indicating significant variability in their amino acid composition compared to other sample types. A visualization of amino acid loadings to assess the relative importance of variables in the sample type submodel reveals compounds such as Asp, Glu, Leu and Val as main contributors of this submodel along PC1, showing a stronger alignment with muscle samples, and to a lesser degree Ala and Ile. Additionally, the score values along PC2 unveil the rest of the differentiation between parasite and liver samples (17%). Ser is highly associated with parasite samples, suggesting significant metabolic activity of Ser in parasites. Conversely, a notable differentiation in Pro compared to other amino acids loadings shows Pro is likely decreased in parasite samples and distinctly enriched in liver and muscle tissues (considering both loading directions). Amino acids were grouped according to their classes, with Phe, Lys, and Tyr clustered together as SAAs, while Ala, Ile, Leu, and Val were grouped as TAAs. However, some TAAs, such as Glu and Pro, were distinct from other TAAs, indicating potentially different metabolic transformations. Ser and Gly were also distinguished from other source amino acids, suggesting unique metabolic behaviors (Fig. 3a). The results of the ANOVA simultaneous component analysis submodel for “infection time” effect has been presented in Fig. 3b. The score plot of first 2 PCs (with 53% and 28% varation, repectively) suggests that the 30-day infection period is notably distinct compared to the others, as it is completely separated from the rest of the days. While, days 90 and 120 exhibit partial separation from each other.

### Trophic dynamics during infection

**Fig. 4:**
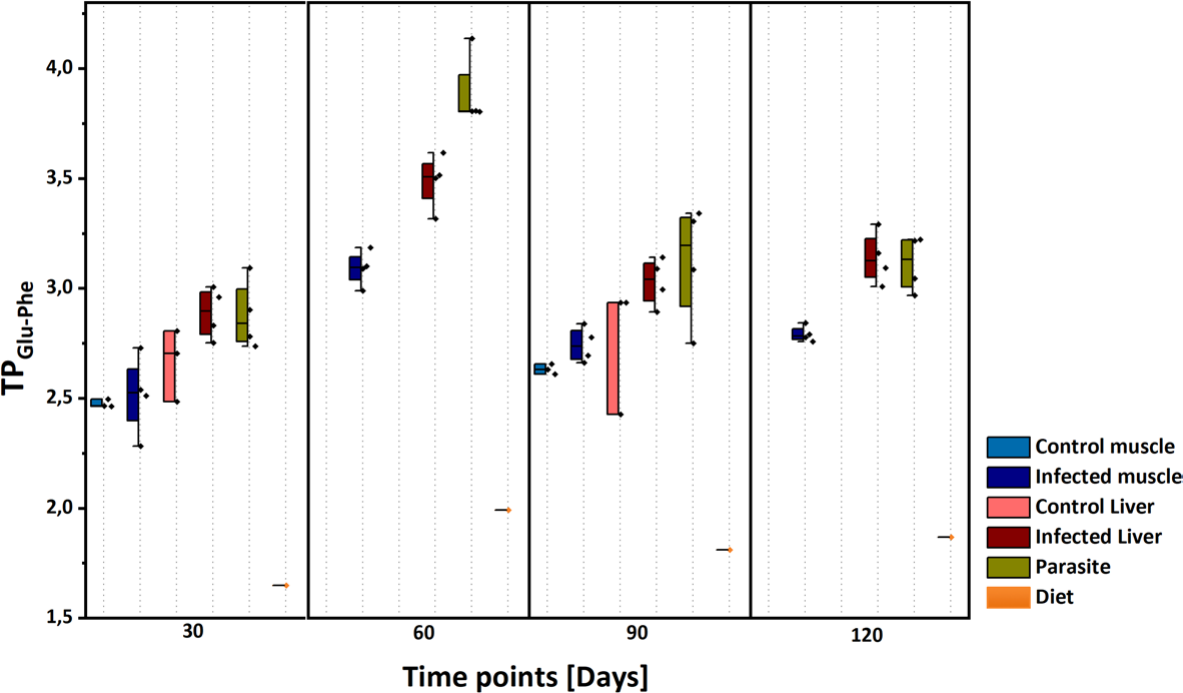
Box plots indicating mean trophic position of host control tissues (n = 3), host infected tissues along with parasite (n = 4) and diet (n = 1) at each time point of 30, 60, 90, and 120 days using conventional method based on Glu as TAA and Phe as SAA.

The mean trophic position for host control tissues (n = 3) was calculated at 30 and 90 days, while for infected host tissues, parasite (n = 4), and diet (n = 1), it was calculated at 30, 60, 90, and 120 days (Table 1). Trophic position was determined using the conventional method described by Chikaraishi et al.^24^, with Glu as the TAA and Phe as the SAA. β (the isotope difference between source and trophic amino acids in the primary producers^24^) values appropriate for freshwater systems, as reviewed by Ramirez et al.^49^, were applied to reflect the environmental conditions in our controlled feeding experiments. Tissue-specific trophic discrimination factors (TDFs) were calculated for both control and infected systems to ensure accuracy in the trophic position assessment (Table 1). A graphical representation of trophic position over time is shown in Fig. 4. The parasite’s mean trophic position closely resembled that of liver tissue, differing by ≤ 0.5 from host muscle trophic position. At 60 days post-infection, an exception was observed where the parasite’s mean trophic position was notably 0.8 higher than that of infected muscle tissue but still 0.5 lower than infected liver tissue. The trophic position difference (∆TP) between infected and control host tissues was minimal, with a maximum difference of ≤ 0.2. Overall, significant changes in trophic position were observed from 30 to 60 days post-infection, with an increase of approximately 1.0 in the parasite and 0.6 in infected host tissues.

**Table 1:** Mean trophic position in control (n = 3) and infected (n = 4) host muscle and liver tissues, along with parasite (n = 4) and diet (n = 1), at 30, 60, 90, and 120 sample collection time points.

TP	Time	Control Muscle		Inf. Muscle		Control Liver		Inf. Liver		Parasite		Diet	
	Point	Avg	SE	Avg	SE	Avg	SE	Avg	SE	Avg	SE	Avg	SE
TP_Glu/Phe_	30	2.5	0.9	2.5	2.3	2.7	1.3	2.9	1.3	2.9	1.4	1.6	1.5
	60			3.1	0.9			3.5	1.1	3.9	1.2	2.0	1.4
	90	2.6	0.9	2.7	1.3	2.8	1.9	3.0	1.4	3.1	1.5	1.8	1.4
	120			2.8	1.0			3.1	1.2	3.1	1.5	1.9	1.5

TDF	Time	Control Muscle		Inf. Muscle		Control Liver		Inf. Liver		Parasite		Diet	
	Point	TDF	SD	TDF	SD	TDF	SD	TDF	SD	TDF	SD	TDF	SD
TDF_Glu/Phe_	30	10.4	0.4	9.6	2.2	7.4	1.0	5.5	1.0	5.6	1.2	7.6	1.2
	60			6.9	0.4			5.1	0.7	4.0	0.9	7.6	1.2
	90	9.8	0.5	8.3	1.0	8.1	1.7	6.0	1.1	5.5	1.3	7.6	1.2
	120			8.3	0.6			5.8	0.9	5.9	1.2	7.6	1.2

The conventional method was used based on two amino acids, with Glu as the TAAs and Phe as the SAA. TDF along with SD in each case is also given. (Inf. = infected; Avg = Average (Mean); SE = standard error; TDF = trophic discrimination factor; SD = standard deviation).

### Δδ^15^N Fractionation between infected and non-infected host tissues

**Fig. 5:**
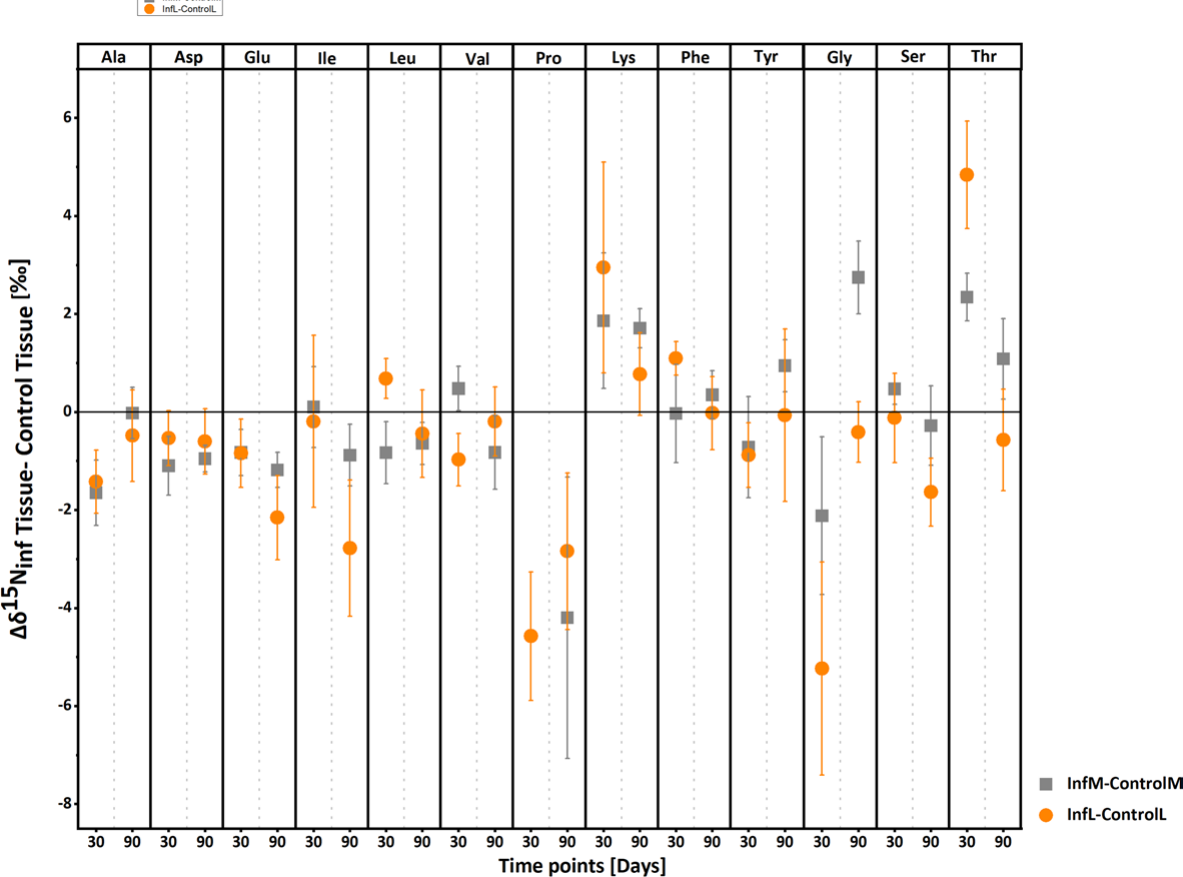
Mean trophic fractionation (Δδ^15^N ± SE in‰) of individual amino acids between infected tissues (n = 4) and control tissues (n = 3); where inf = infected, M = host muscle tissue and L = host liver tissue.

Trophic fractionation of amino acids, denoted as Δδ^15^N_Infected Tissue–Control Tissue_, calculated to assess differences between infected (muscle and liver; n = 4) and control tissues (muscle and liver; n = 3) at sampling days 30 and 90. Results include the standard error (SE) of the difference between means (Table S4) and are presented in Fig. 5. In comparing infected and control tissues, a general decrease in Δδ^15^N values was observed for TAAs. Proline exhibited the most significant decrease, with Δδ^15^N_InfL–ControlL_ of − 4.6 ± 1.3‰ in infected liver relative to control liver at 30 days post-infection, and Δδ^15^N_InfM–ControlM_ of − 4.2 ± 2.9‰ in infected muscle relative to control muscle at 90 days post-infection. Glutamic acid and Ile also showed decreases exceeding 2‰ in infected liver tissue compared to control liver at 90 days post-infection. Conversely, SAAs and MAAs generally showed increases in infected muscle tissue. Glycine had the most pronounced increase with a Δδ^15^N_InfM–ControlM_ of 2.7 ± 0.7‰ at 90 days post-infection, followed by Thr at 2.3 ± 0.5‰ at 30 days post-infection relative to control muscle tissue. However, in infected liver tissue, Gly, Ser, and Tyr exhibited decreased Δδ^15^N values compared to control liver tissue. In contrast, Thr, followed by Lys, showed significant increases, with Δδ^15^N_InfL–ControlL_ values of 4.8 ± 1.1‰ and 2.9 ± 2.1‰, respectively, at 30 days post-infection in infected liver relative to control liver tissue. Among SAAs, Phe showed the least fractionation in both tissues.

## Discussion

In this study, we explored the interactions between diet and consumers, focusing on the amino acid δ^15^N values that highlight the metabolic pathways connecting the parasite *Schistocephalus solidus* and its three-spined stickleback host (*Gasterosteus aculeatus*). Our findings reveal three distinct categories of amino acids, highlighting the dynamic relationship between consumer tissues and their dietary sources. Notably, the pronounced trophic fractionation observed between host tissues and diet not only underscores the liver’s higher turnover rate but also points to a critical metabolic link between the liver and the parasite, as evidenced by similar shifts in nitrogen isotope turnover. Through a carefully designed controlled feeding experiment, we investigated these connections across multiple tissues at various time points, calculating trophic positions to illustrate the direct assimilation of host-derived nutrients by the parasite. These insights not only enhance our understanding of the metabolic connections within host-parasite interactions but also underscore the importance of considering multiple host tissues in future ecological and physiological studies.

As we delve into the discussion, we will elucidate the implications of these findings, supported by relevant examples from our analyses. Our results from diet-consumer interactions indicate three categories of amino acids based on fractionation between consumer tissues (control and infected liver and muscle tissue along with parsite) and diet. The results demonstrate that TAAs undergo significant isotopic enrichment during trophic transfer, attributed mainly to deamination^50^, whereas SAAs exhibit minimal enrichment, indicating stable metabolic processes^22^. Threonine poses a classification challenge due to its different nitrogen isotopic values, leading to its reclassification as a metabolic amino acid, which signifies its distinct metabolic pathway compared to other source amino acids^51,52^. However, the behavior of Pro and Ser in parasite tissue did not align with these general amino acid categories, indicating unique metabolic pathways for these amino acids within the parasite. Fractionation between consumer tissues (infected and control systems) and diet (Δδ^15^N_consumer Tissue–Diet_) shows an increase in δ^15^N values of consumer tissues up to 90 days, suggesting effective nitrogen assimilation from the diet, resulting in higher nitrogen isotope fractionation in tissues relative to the diet. The subsequent decrease in fractionation from 90 to 120 days likely indicates a stabilization in isotopic composition, suggesting the system may be reaching an equilibrium state. This equilibrium may imply that the host tissues are no longer accumulating nitrogen at the same rate, possibly due to metabolic saturation or a shift in metabolic demand. The minimal change (< 0.5‰) in δ^15^N values for specific amino acids (Ala, Glu, and Tyr) in muscle tissue between 90 and 120 days suggests that muscle tissue may have achieved a stable state with limited fluctuation in nitrogen assimilation. This result aligns with previous research, which indicates that muscle tissue, due to its slower turnover rate, acts as a more conservative indicator of long-term dietary intake, showing less response to short-term isotopic changes in the diet^41^. Conversely, the more dynamic shifts in Δδ^15^N values observed in liver tissue across all time points imply a higher turnover rate or increased metabolic activity, consistent with the liver’s essential role in metabolism and nutrient processing^53^. As Carter et al.^54^ describe, fast-turnover tissues like liver and plasma often reflect recent dietary changes, while slower-turnover tissues such as muscle and bone are influenced by longer-term diet trends. In addition, the trophic fractionation (Δδ^15^N_Para-diet_) of most amino acids in parasite tissue displayed a similar turnover pattern to that of infected liver tissue relative to diet, indicating comparable nitrogen assimilation dynamics between the parasite and liver tissue. This similarity in turnover pattern suggests a close metabolic relationship between parasite and liver, a connection also observed by Hesse et al.^41^ in a controlled feeding experiment using δ^13^C amino acid stable isotope analysis.

In the complex realm of fish host-parasite interactions, the stable isotope values of parasites display significant variability, reflecting a complex interaction of factors. Parasites like cestodes and acanthocephalans absorb nutrients through their tegument, taking up host-derived compounds such as amino acids, peptides, and carbohydrates^11^. These metabolites are typically depleted in heavier isotopes due to kinetic isotope effects favoring lighter forms during biochemical reactions^55^. As a result, cestodes often exhibit lower nitrogen isotope values compared to their hosts^11,56,57^. This depletion varies depending on the parasite’s feeding location and the host’s nutritional condition, leading to isotopic differences among parasite populations and tissues^48^.

In terms of δ^15^N-AA values , parasites typically had lower values for most SAAs compared to host tissues, reflecting a conventional pattern of passive feeding behavior. However, exceptions arise with Ser, which has values higher in parasites compared to both host tissues, and Thr, which were higher in *S. solidus* compared to host liver tissue. During the development of plerocercoids the Gly values decreased over time in parasites, suggesting metabolic transformation. Gly, Ser, and Thr take part in common metabolic pathways involving enzymes such as Thr/Ser ammonia-lyase, Ser hydroxymethyltransferase, and the Gly cleavage system^58,59^. These pathways likely contributed to the observed fractionation of Ser and Thr and negative fractionation of Gly. Similar fractionation patterns of these three amino acids were reported also for carbon in the same stickleback- *S. solidus* host-parasite system^10^. This outcome suggest the liver as a hub of metabolism, playing a significant role in nutrition transfer between the host and parasite, with notable metabolic activities observed in liver tissues^35^. Glycine, Serine, and Threonine participate in interconnected pathways involving enzymes such as Thr/Ser ammonia-lyase^60^, Ser/Gly hydroxymethyltransferase, and the Gly cleavage system^61^, which likely contributed to the observed fractionation: positive for Ser and Thr, and negative for Gly. Unlike Thr, Ser can also be produced through endogenous synthesis, where the balance between de novo synthesis and dietary intake affects measured δ^15^N of Ser results^62^, potentially leading to higher Δ^15^N fractionation of Ser compared to other amino acids. The atypical Δ^15^N increment observed in parasites may be attributed to unique metabolic processes specific to each parasite taxon, as indicated by studies suggesting that Δ^15^N variations in parasite-host pairings could be linked to the phylogenetic histories of parasites^5,63^.

Among TAAs, Glu and Pro exhibit distinctly lower δ^15^N values relative to other amino acids when comparing the parasite to host liver tissue. Notably, Pro shows the highest degree of fractionation (Δδ^15^N_P-L_ =  ~ − 5‰, Δδ^15^N_P-M_ =  ~ − 8‰), consistent with previous research^51,52,64^, across all amino acids in comparisons between the parasite and both host tissues.

The high fractionation suggests a potential conversion of Pro to hydroxyproline, a post-translational modification requiring vitamin C that is vital for parasite collagen-like structural development^35^. Such conversion may deplete Pro in parasite tissues and result in lowered δ^15^N values, with the host’s higher Pro concentrations acting as a reservoir. Previous research suggests that hydroxyproline supplementation can enhance growth rates in fish^65^, which may provide insights into similar growth mechanisms in parasites. Furthermore, decreased Δ^15^N values of Pro and Glu compared to other TAAs could result from the conversion of Glu to Pro to facilitate these metabolic activities. Proline cannot undergo transamination because its secondary amino group is part of a ring structure^23^. However, the amino-nitrogen of Pro, as well as hydroxyproline, is derived from the same nitrogen pool as the α-nitrogen of Glu, because Pro is synthesized from Glu via ring closure^66^. The interaction of nitrogen among transaminating amino acids and Pro, including post-translationally modified forms such as hydroxyproline, complicates the interpretation of nitrogen isotopic values in consumer tissues^23^. It challenges the conventional assumption that the nitrogen isotopic values of TAAs in a consumer solely reflect the nitrogen isotopic value of those amino acids in the diet with an offset due to metabolism^27,67,68^. Instead, there is an averaging effect due to the extent of nitrogen cycling through the metabolic nitrogen pool. This effect arises from nitrogen cycling through the metabolic pool, resulting in the diet’s averaged isotopic signal of all transaminating amino acids^23^. Furthermore, the isotopic signal of any specific amino acid in a particular body tissue or pool is also influenced by various other factors specific to the individual^23^. These factors include metabolic and physical compartmentation between and within tissues^69,70^, the equilibrium between amino acid synthesis and oxidative degradation^70^, the scavenging of urea from the gastrointestinal tract^71^, and the methods and paths of nitrogen elimination^29,51^. These complexities highlight the complex dynamics of nitrogen metabolism and its significance in interpreting isotopic information in ecological research.

The increase in δ^15^N values of Ala, in parasites by up to 5‰ over a 90-day period suggests its involvement in parasitic growth. As an essential precursor of gluconeogenesis, Ala undergoes deamination^72^, resulting in an increased δ^15^N value due to the preferential removal of the lighter nitrogen isotope. The metabolic processes such as transamination and deamination, which are prevalent in the metabolic pathway for TAAs, typically favor the lighter stable isotope (^14^N-containing amine groups;^73^ through kinetic fractionation^29^). Moreover, the glycogen content in cestode plerocercoids suggests the necessity of glucose biosynthesis from other nutrients^74^, highlighting the elaborate metabolic processes involved in parasite-host interactions.

Amino acid CSIA is an effective tool for detecting subtle variations in trophic positions within parasite-host systems. By examining both the direct uptake of compounds by the parasite and the host’s dietary nitrogen sources, amino acid CSIA offers insight into trophic dynamics within the food web^75^. Using the conventional method^24^, ΔTP between parasite and host generally remains below 0.5, consistent with observations in cestodes, where parasite δ^15^N values show relatively little variation compared to their hosts^63^. This phenomenon is attributed to direct compound assimilation through a syncytial, ‘microtrich’-covered tegument^47^ instead of host tissue digestion and metabolism^48^. An exception arises at 60 days post-infection, where ΔTP between parasite and muscle tissue increases to 0.8, although it remains lower (within 0.5) than liver tissue. Overall, trophic position values are stable across time points for both host tissues and the parasite, with a notable shift occurring at 60 days post-infection. Here, ΔTP between infected host tissues and the parasite exceeds 0.5‰, while the parasite itself shows a pronounced shift in trophic position by up to 1. This pattern may suggest that during periods of heightened host metabolism or parasite growth, the parasite assimilates ^15^N-enriched Glu more actively. Given that Glu is central to nitrogen metabolism^76^ and essential for amino acid transamination via glutamate dehydrogenase^77^, this shift may reflect increased nitrogen flux in host tissues. The observed differences between liver and muscle tissue may also play a role. Liver tissue, which typically has a faster nitrogen turnover rate and greater metabolic activity than muscle^35^, more rapidly incorporates dietary nitrogen and thus aligns more closely with short-term isotopic shifts.

When comparing the infected host’s muscle and liver tissues with control tissues, the Δδ^15^N_Infected Tissue- Control Tissue_ values of TAAs showed slight decrease, except for Pro. This is reflected in the trophic position of both groups, which were only slightly different, indicating similar TLs for infected and control hosts. Among other amino acids, Gly and Thr exhibited prominent trends over the course of the 60-day infection period, from 30 to 90 days post-infection, suggesting their significant involvement in the host’s metabolic response. Infections by helminth parasites often activate the host’s immune system^78^, leading to increased protein synthesis and cell proliferation. Gly, which functions as immunomodulatory agent, anti-inflammatory, and a neurotransmitter, plays a crucial role in preventing endotoxic shock, sepsis, and apoptosis^79^. Its increase over time may reflect the host’s increased metabolic demand for immune support during infection. Additionally, Gly is essential for collagen synthesis and immune function^80^, so its retention and utilization during infection may be linked to tissue repair and immune responses. This aligns with findings from Hesse et al. (2023)^10^, who observed a similar trend in δ^13^C values in the same system, where Gly played a prominent role in distinguishing infected vs. uninfected sticklebacks.

The growth of parasites places significant metabolic demands on the host, as it must not only provide nutrients for its own maintenance but also to fuel the parasite’s development^10^. In response, the host may catabolize specific amino acids such as Thr and Gly. The breakdown of Thr yields Gly and acetate, both vital for cellular metabolism^81^. The elevated δ^15^N values of Thr in infected tissues likely reflect increased catabolic activity, while the resulting Gly shows lower δ^15^N values due to its isotopically lighter composition. Moreover, this increased amino acid fractionation was more pronounced at 30 days post-infection than at 90 days post-infection, indicating stronger metabolic effects during the early stages of infection. Scharsack et al. (2007)^42^ similarly reported higher infection prevalence (> 60%) during early stages (7–17 days) versus later stages (45–52% at 27–67 days), with parasites growing ~ 17-fold early on. This finding is further confirmed by our ANOVA simultaneous component analysis model, which identified infection as a significant factor driving amino acid fractionation, particularly at 30 days post-infection. Compared to muscle, liver tissue showed greater shifts, suggesting parasites may rely on liver-derived metabolites rather than breaking down host tissue. This is supported by prior finding^42^ of liver shrinkage in infected hosts, emphasizing the liver’s central role in nutrient allocation during infection.

## Conclusion

Our study provides a detailed insights into the nitrogen isotope signatures of individual amino acids in the parasite *S. solidus* and its three-spined stickleback host, emphasizing the metabolic pathways in the host-parasite system. By analyzing both liver and muscle tissues from the host, we found that the parasite’s metabolic activity is more closely linked to the host’s liver tissue. A significant enrichment of 5‰ in Ala in the parasite over 90 days indicates its important role in metabolic pathways, particularly gluconeogenesis, which is essential for parasite growth. The minimal ΔTP between the parasite and host tissues, averaging below 0.5, suggests that the parasite directly assimilates host-derived nutrients. This highlights the close metabolic connection, particularly with the host liver, as evidenced by the notable enrichment of amino acids such as Ser, Gly, and Ala, which play critical roles in gluconeogenesis. The findings underline the significance of the liver’s higher turnover rate compared to muscle tissue, revealing a crucial connection in nitrogen assimilation dynamics between the host’s liver and its parasitic counterparts. Proline, on the other hand, displayed distinct metabolic behavior, highlighting the complexity of the host-parasite metabolic interactions. Additionally, we observed an increasing trend of Gly in infected host tissues over time compared to control tissues, which likely reflects the host’s immune response to infection. These findings emphasize the importance of studying multiple host tissues to fully understand the complex metabolic interactions between host and parasite. While multivariate analysis identifies general patterns of δ^15^N values across tissue types, we recommend future studies using ^15^N-enriched amino acids in controlled feeding experiments. This will allow for more precise tracking of metabolic pathways and provide a deeper understanding of nutrient exchange and energy flow in the host-parasite system, offering a more comprehensive view of their ecological and physiological interactions.

## Materials and methods

### Infection and feeding experiment

As part of a parasitic infection experiment three-spined Sticklebacks were laboratory-raised offspring and reared in twelve 14 L tanks (VewaTech, Germany). The water was recirculated and held at 18 °C with a 15 h light and 9 h dark cycle. Stickleback offspring were produced by in vitro fertilization from individuals collected from a brook in North-West Germany (52°17′33.11′′ N, 7°36′46.48′′ E), about eight months old at the beginning of the experiment and fed daily with washed red mosquito larvae (*Chironomidae*) over four months.

**Fig. 6:**
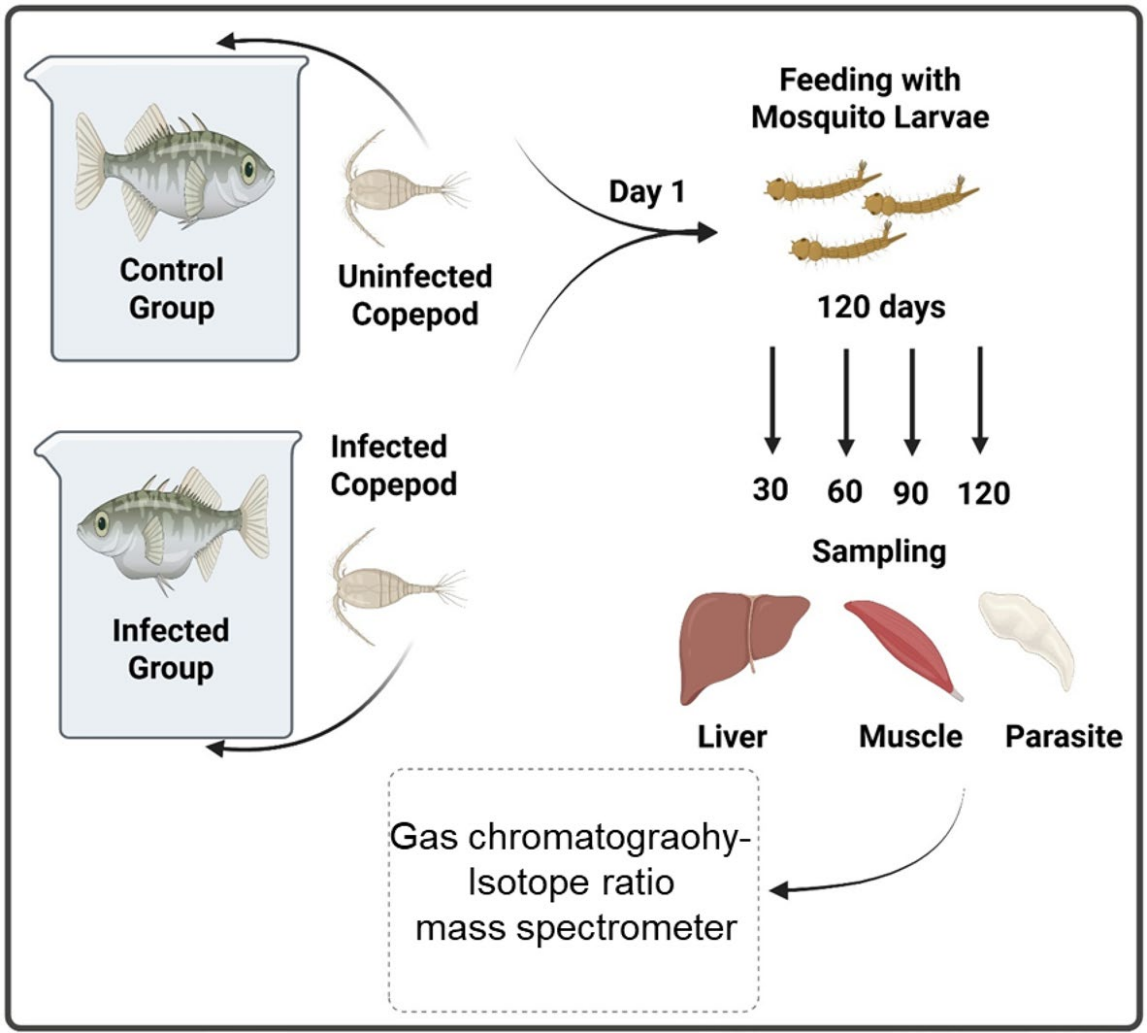
Schematic diagram of the experimental setup used to study amino acid nitrogen isotope fractionation in a parasite-host system. Sticklebacks were divided into two groups: control fish were fed with uninfected copepods, while the infected group received copepods harboring cestode larvae. All fish were additionally fed mosquito larvae throughout the experiment. Sampling was conducted at 30, 60, 90, and 120 days post-infection. At each time, liver and muscle tissues from both control and infected fish were collected, along with developing parasites from infected hosts. Tissues were analyzed using Gas chromatography-isotope ratio mass spectrometer to assess nitrogen isotope fractionation and trophic interactions over time.

The samples used in this study were obtained from the same controlled infection/feeding experiments employed in a prior investigation^41^. Briefly, stickleback and parasites were raised under controlled laboratory conditions. For the infection/feeding experient, sticklebacks were individualized in jars and fed with a single infected copepod. Sham exposed controls received a copepod without parasite. On the next day, ingestion of copepods was confirmed and fish were returned to their experimental tanks. Feeding and host/parasite growth was continued up to 120 days. A slight modification from Hesse et al.^41^ was made in the sample collection process: instead of five, only four sham-exposed individuals per date (30, 60, 90, and 120 days post-infection were used, with liver and muscle tissues of the infected host being subjected to CSIA. Additionally, we employed pooled dietary samples of mosquito larvae, consistent with the methodology outlined in the study^41^. Specifically, washed red mosquito larvae (Chironomidae) served as a controlled dietary source for the sticklebacks. To assess the nutritional composition over time, subsamples of the mosquito larvae were collected weekly and stored at − 20 °C. These subsamples were subsequently pooled into four time intervals: 1–30 days, 31–60 days, 61–90 days, and 91–120 days, for CSIA. Dried red mosquito larvae are commercially available and provide a protein-rich diet for fish, containing up to 60% crude protein and approximately 5% crude lipid. While the amino acid profile of these larvae is generally balanced for aquaculture, it is important to note that certain amino acids, such as histidine (His), Lys, or tryptophan (Trp), may be deficient depending on the specific insect species used^82,83^. For ethical compliance, sticklebacks were starved for 72 h prior to sampling, anesthetized using MS 222 (Sigma-Aldrich, USA), and killed by decapitation. Liver and muscle tissues were carefully collected, excluding skin and bones, and stored at − 20 °C until analysis. The maintenance and handling of sticklebacks adhered to animal welfare standards established by the EU Directive 2010/63/EU for animal experimentation. Approval for all animal procedures was obtained from the ‘State Agency for Nature, Environment and Consumer Protection’ (LANUV) of North Rhine-Westphalia (Project No. 87 51.04.2010.A297), following an ethical review. This study was also conducted in accordance with ARRIVE guidelines (https://arriveguidelines.org/) for transparent reporting in animal research. For the controlled samples (fish hosts without infection), only three individuals were selected for δ^15^N analysis at 30 and 90 days post-infection to accommodate any potential changes that may arise. A schematic overview of the experimental design, including feeding regimes, infection status, and tissue sampling time points, is provided in Fig. 6 to facilitate understanding of the study’s workflow.

### δ^15^N compound specific isotope analysis of amino acids

Samples were prepared and analyzed following the method of Riekenberg et al.^84^, and normalized following Yarnes and Herszage 2017^85^ at the Marine Microbiology and Biogeochemistry Department, NIOZ Royal Netherlands Institute for Sea Research (Texel). For CSIA of amino acids, dried and homogenized tissue (2–5 mg) was lipid extracted, acid hydrolyzed, derivatized to pivaloyl–isopropyl esters and analysed for δ^15^N of amino acids via gas chromatography–combustion isotope mass spectrometry following the method presented in Riekenberg et al.^84^. Nitrogen isotope analysis values for 14 amino acids are reported: Ala, Asp, Glu, Gly, Leu, Lys, Ile, Met, Phe, Pro, Ser, Thr, Tyr and Val when possible. Precision of δ^15^N-AA values were < 0.5‰ in standards run 10 times per sequence for 14 amino acids throughout the analytical runs supporting this study and sample duplicates had an mean precision of 0.24‰. While δ^15^N values were obtained for up to 14 amino acids, our interpretation focused on those most biologically informative for addressing trophic dynamics and host-parasite metabolic interaction (see supplementary Fig. S1). Amino acids such as Glu, Phe, Ala, Pro, Ser, Thr, and Gly were prioritized based on their roles in trophic position estimation, known metabolic significance, and distinctive isotopic patterns across infection stages.

### Trophic position calculation based on tissue specific TDF

The trophic position of both control and infected host tissues, as well as the parasite, was determined using the conventional method outlined by Chikaraishi et al.^24^. Trophic position was calculated using Chironomid (Diptera: Chironomidae) larvae as the diet, which exhibit diverse modes of life and feeding habits, representing nearly all feeding groups and playing a crucial role in various aquatic ecosystems^86^. Therefore, in this study, trophic position was calculated using β values based on freshwater systems to appropriately reflect the ecological conditions relevant to the aquatic environments used in our controlled feeding experiments. In this method, Glu was designated as the TAA, while Phe was used as the SAA. The trophic position for Glu/Phe (TP _Glu/Phe_) was calculated using the following equation:

1 $${\text{TP}}_{\text{Glu}/\text{Phe}} = 1 + \frac{{\delta }^{15}{N}_{Glu}-{\delta }^{15}{N}_{Phe}-{\beta }_{Glu/Phe}}{{TDF}_{Glu-Phe}}$$

where β_Glu/Phe_ = 4.2 ± 0.7‰, based on the review by Ramirez et al.^87^, given that the mosquito larvae were fed freshwater microalgae. The tissue specific TDF _Glu–Phe_ (Table 1) was calculated based on ^15^N-enrichment factor (∆) of Glu and Phe as follows (Eq. 2):

2 $${\text{TDF}}_{{{\text{Glu}} - {\text{Phe}}}} = \Delta _{{{\text{Glu}}}} {-} \Delta _{{{\text{Phe}}}}$$

where ∆ values for Glu and Phe were derived using equation (Eq. 3)^24^:

3 $$\Delta = \delta ^{{{\text{15}}}} {\text{N}}_{{{\text{Consumer}}}}{-}\delta ^{{{\text{15}}}} {\text{N}}_{{{\text{Diet}}}}$$

Here, the “Consumer” refers to the control host tissues, infected host tissues, and parasite. Tissue-specific Δ values for all amino acids are provided in Tables S5 and S6.

### Data analysis

Data analysis was conducted using Excel from Microsoft Office 365 ProPlus (Microsoft, Redmond, Washington, USA), Origin pro 2022 (OriginLab, Northampton, Massachusetts, USA) and Matlab 9.10 R2021a (TheMathWorks, Natick, MA, USA). Isotope data are presented as mean δ^15^N values for each amino acid in infected and control samples at individual time points on the international reference scale in per mill (‰), accompanied by their corresponding standard deviations (SD) in the supplementary material (Tables S1, S2 and Fig. S2). Isotope ratios are generally expressed in the δ-notation. The general expression is provided in Eq. 4.

4 $${\delta \text{X}}=\left[\left(\frac{{\text{R}}_{\text{sample}}}{{\text{R}}_{\text{std}}} \right) -1\right] \times 1000$$

where, X represents the heavy isotope form of an element (e.g., ^15^N), R signifies heavy-to-light isotope ratio (e.g., ^15^N/^14^N), and values are expressed in per mil (‰). For nitrogen, the international standard utilized is atmospheric nitrogen (AIR; δ^15^N).

Trophic fractionation (Δδ^15^N) of amino acids between host control tissues (n = 3) and the diet (n = 1) was calculated at sampling days 30 and 90, with the SE of the mean difference provided in Table S5 and illustrated in Fig. 1a. For infected system, fractionation ± SE between muscle, liver, and parasite tissues (n = 4) and diet was assessed on sampling days 30, 60, 90, and 120 (Table S6, Fig. 1b). To evaluate the mean trophic fractionation between parasite and infected host tissues (n = 4) over 120 days post-infection, paired sample t-tests were conducted against zero (DF = 15, α = 0.05). Additionally, multivariate statistical approach using ANOVA simultaneous component analysis^88^ was applied to the data matrix of δ^15^N values of amino acids, from infected sticklebacks and parasites, by using PLS Toolbox 8.9.1 (Eigenvector Research Inc., Wenatche, WA, USA). ANOVA simultaneous component analysis model is especially useful in studies with limited sample sizes because it helps clearly separate the effects of different experimental factors from random variation in the data^88^. Moreover, to strengthen inference, we performed 10,000 permutations of factor labels in the ASCA framework to build an empirical null distribution of the sum‐of‐squares and derive p-values based on this non-parametric test without relying on distributional assumptions. In this ANOVA simultaneous component analysis model, the effects of three categorical factor (sample types of muscle, liver, and parasite, set as F1) and sampling time (30, 60, 90 and 120 days post-infection, set as F2) and interaction were evaluated, through a well-balanced design matrix. Statistical significances of the two factors and interaction were assessed by a permutation test, using 10,000 permutations^89^. The mean trophic position (using conventional method^24^) for host control tissues (n = 3) was calculated at 30 and 90 days. In contrast, for infected host tissues and parasites (n = 4), as well as the diet (n = 1), trophic position was calculated at 30, 60, 90, and 120 days (Table 1), with results presented in Fig. 4. Tissue specific TDF was calculated in both control and infected system (Table 1). Trophic fractionation (Δδ^15^N) of amino acids between infected liver/muscle tissues (n = 4) and control liver/muscle tissues (n = 3) was also calculated for sampling days 30 and 90, along with the SE of the mean difference, as shown in Table S4 and illustrated in Fig. 5.

## Supplementary Information

Below is the link to the electronic supplementary material.

Supplementary Material 1

## Data Availability

The datasets generated during and/or analysed during the current study are available in the Figshare repository: https://figshare.com/s/6452d8c54341ba5386ae
