# Supplementary material for: Trophic dynamics and metabolic pathways in host parasite interactions revealed by nitrogen isotope analysis of amino acids in multiple tissues
Source: Sci Rep. 2025 Sep 18;15:32600. doi: 10.1038/s41598-025-19052-0 (PMC12446447; doi:10.1038/s41598-025-19052-0)
Supplement: Supplementary file 1 — Supplementary Material 1 [file 41598_2025_19052_MOESM1_ESM.docx]

**Appendix Information**

**Trophic Dynamics and Metabolic Pathways in Host-Parasite Interactions: A Nitrogen Isotope Analysis of Amino Acids in Multiple Tissues**

Shaista Khaliq^1,3^, Milen Nachev^2,3^, Philip M. Riekenberg^4,5^, Maik A. Jochmann^1,3*^, Maryam Vosough^1,3^, Frederik Franke^7,9^, Jörn P. Scharsack^7,8^, Joachim Kurtz^7^, Bernd Sures^2,3,6^, Marcel T.J. van der Meer^4^, Torsten C. Schmidt^1,3^

1. Instrumental Analytical Chemistry, University of Duisburg-Essen, Universitätsstr. 5, 45141 Essen, Germany
2. Aquatic Ecology, University of Duisburg-Essen, Universitätsstr. 5, 45141 Essen, Germany
3. Centre for Water and Environmental Research, University of Duisburg-Essen, Universitätsstr. 5, 45141 Essen, Germany
4. Marine Microbiology and Biogeochemistry Department, NIOZ Royal Netherlands Institute for Sea Research, 1790AB, Den Burg, the Netherlands
5. Center for Applied Isotope Studies, University of Georgia, Athens, GA 30605
6. Research Center One Health Ruhr, Research Alliance Ruhr, University of Duisburg-Essen, 45141 Essen, Germany
7. Institute for Evolution & Biodiversity, University of Münster, Hüfferstr. 1, 48149 Münster, Germany
8. Present address: Thünen Institute of Fisheries Ecology, Herwigstr. 31, 27572 Bremerhaven, Germany
9. Present address: Bavarian State Institute of Forestry, Hans-Carl-von-Carlowitz-Platz 1, 85354 Freising, Germany

**Sample preparation and analysis**

**Hydrolysis and derivatization:**

Sample hydrolysis and derivatization were carried out according to the method by Riekenberg (2020). About 2 mg of freeze-dried and homogenized tissue was placed in a 1 mL Supelco reaction vial (Darmstadt, Germany) covered with PTFE tape. Then, 200 µL of 6 M HCl was added, and vials were capped with a mininert 15 mm valve cap (Supelco). Positioned on a dry heating block at 110°C with pH indicator paper on top, vials were closed upon indicating acidity for oxygen purging. Vials were heated at 110°C overnight for acid hydrolysis. After hydrolysis, samples cooled, and acidified water was transferred to a GHP Nanosep centrifuge filter of 0.45 µm (Medemblik, Netherlands) and centrifuged (2 minutes, 3,000 RPM). For lipid removal, the liquid was transferred to a clean vial, 300 µL of n-hexane: DCM (3:2) was added, and the mixture was capped. After centrifugation for a few seconds, allowing water and organic solvent separation, the top organic layer was pipetted off and discarded. This purification step was repeated twice. For internal calibration, a norleucine (Nle) reference spike (44.8 mM in 0.1 M HCl) was added to all samples and standards post-hydrolysis, following the method by Yarnes and Herszage (2017)^1^. The derivatization method used was N-pivaloyl/isopropyl (NP/iPr), a technique commonly applied in nitrogen isotope analysis of amino acids^2-5^. Isopropylation involved adding 300 µL of organic solvent mixture of isopropanol and acetyl chloride (1:4) and heating at 110°C for 2 h. Valves on vial caps were unsealed initially to flush out oxygen. After cooling, the mixture was evaporated under N_2_ at 40°C. Dichloromethane (DCM; 150 µL) was added and evaporated to dryness to remove reagents and this process is repeated twice. Isopropyl esters were acylated with 300 µL of pivaloyl chloride and DCM (1:4) at 110°C for 2 h. After cooling, the reagent was evaporated under N_2_ at 40°C, and 150 µL of DCM was added twice to help remove excess regaents. Then, around 5 drops of water and 150 µL n-hexane: DCM (3:2) were added, shaken, and the organic phase transferred to a MgSO_4_ column. After repeating the addition/removal and rinsing steps, the organic fraction was evaporated to dryness under N_2_ at 40°C. Ethyl acetate was added, and the sample stored at -20°C for GC-IRMS analysis.

Amino acids (L-form) of >98% purity were purchased from Sigma Aldrich (Darmstadt, Germany) and Arndt Schimmelmann (Indiana University Bloomington, USA) and used for in-house reference standard mixtures. The "scaling AA mix," used for scale normalization, included L-norleucine (Nle) and five amino acids with known δ^15^N values. This mixture (~44 mM) prepared in 0.1 M HCl, consisted of AAs- Ala, Gly, USGS65; USGS, Reston VA, USA), Val, Glu, and Phe. For calculating offsets during derivatization and combustion, the "offset AA mix" comprised 14 amino acids and the internal reference standard Nle. This standard mixture (~8.8 mM) prepared in 0.1 M HCl, including amino acids such as Ala, Gly, Val, Ile, Leu, Pro, Asp, Thr, Ser, Met, Glu, Phe, Lys, and Tyr. An internal reference spike of Nle (44.8 mM), prepared in 0.1 M HCl, was used for inclusion into samples after acid hydrolysis. Storage for all three solutions was in the dark at −20°C. High purity solvents and reagents used during acid hydrolysis and derivatization, including acetyl chloride (Purity: >99%, Fluka, Seelze, Germany), trimethylacetyl chloride (Purity: >98%, Alfa Aesar), bi-distilled water, anhydrous magnesium sulfate (MgSO_4_) (Purity: ≥ 99.5%, Alfa Aesar, Waltham, MA, USA), hydrochloric acid (VWR International, Boxmeer, The Netherlands), and HPLC-grade dichloromethane (DCM), hexane, methanol, ethyl acetate, and isopropanol (Promochem, Berlin, Germany).

**GC-IRMS analysis:**

The analyses were performed using a Trace 1310 gas chromatograph (GC), linked to a Delta V Advantage irMS instrument through a GC IsoLink II combustion interface. The whole instrument was supplied by Thermo Fisher Scientific, Bremen, Germany. The oxidation-reduction reactor used a NiO tube filled with CuO/NiO/Pt wires inside an aluminum oxide tube, which was consistently maintained at 1000°C during operation. The NP/iPr AA derivatives, dissolved in solvent (ethyl acetate), underwent injection at approximately 75°C using a PTV for on-column injection, ramped alongside a glass column. The separation occurred on a DB-5MS column (60 m × 0.32 mm × 0.5 µm; Agilent Technologies, USA) with a constant flow rate of 2.0 mL min^-1^. The initial column oven temperature was held at 70°C for 1 min, increased to 165°C at 50°C min^-1^, then increased to 185°C at 2°C min^-^1 and finally increased to 300°C at 10°C min^-1^, and held for 7 min. A ~1.5 m pre-column (deactivated fused silica) of 0.53 mm was used before the analytical column to provide protection. Contaminated sections of the pre-column were regularly cut off, or the entire pre-column was replaced approximately every second or third sequence to counter peak tailing from material buildup. Using these parameters with GC-IRMS, 15 AAs including Nle were separated (Fig.1).

**Fig S1:** GC-IRMS δ^15^N isotope analysis of standard mixture (~8.8 mM) of 15 amino acids including L-norleucine (Nle) which is used as an internal standard. (Traces for m/z 29 green and m/z 29 blue line are shown.)

**Figure S2:** δ^15^N (‰ ) along with SD of AAs of control host tissues (n=3) at 30 and 90 days and infected host tissues along with parasite (n=4) and diet ((n=1) at 30, 60, 90 and 120 DPI. Upper graph indicates TAAs and lower indicates SAAs. All sample types can be differentiated by different colours.

**
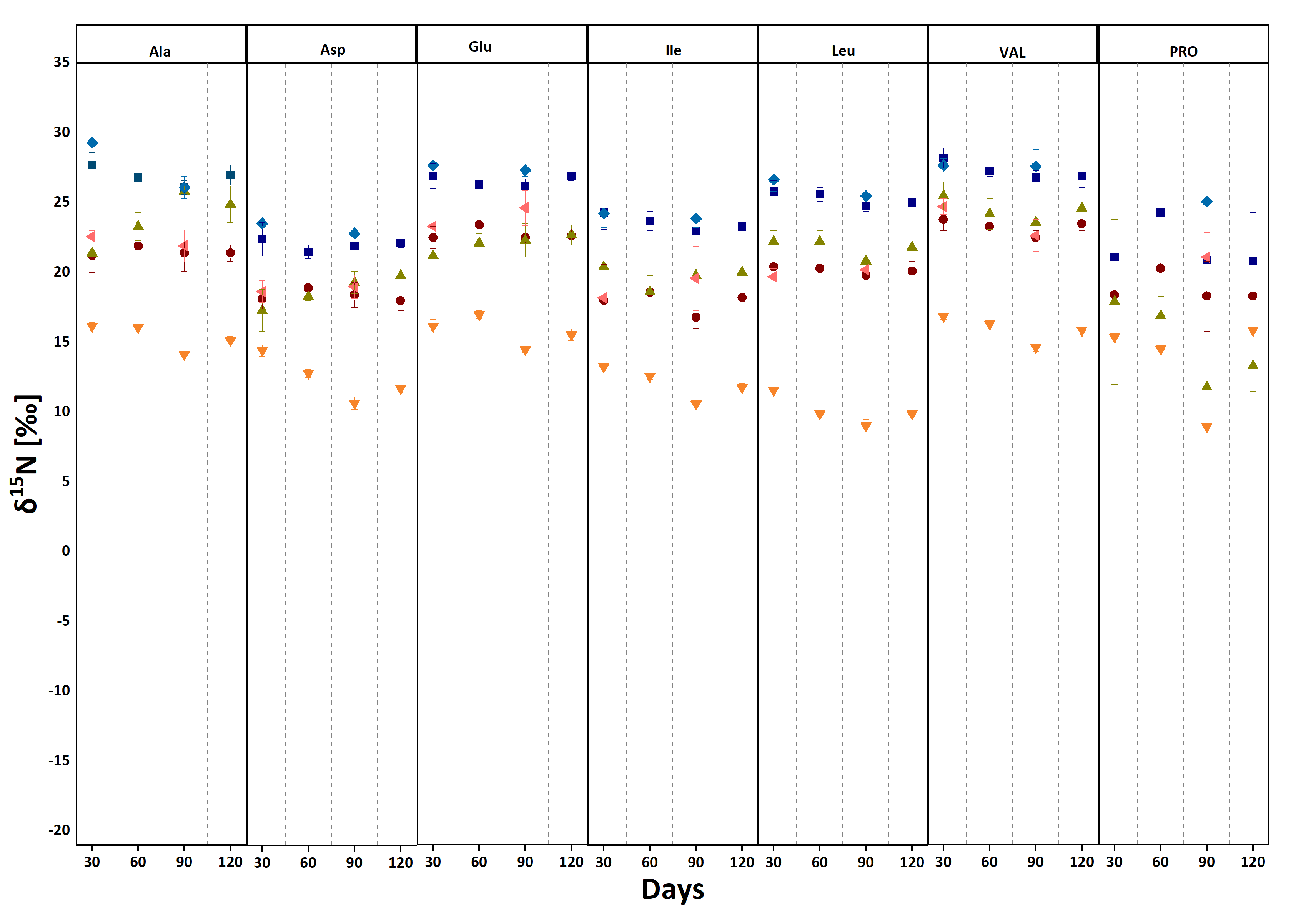
**

**
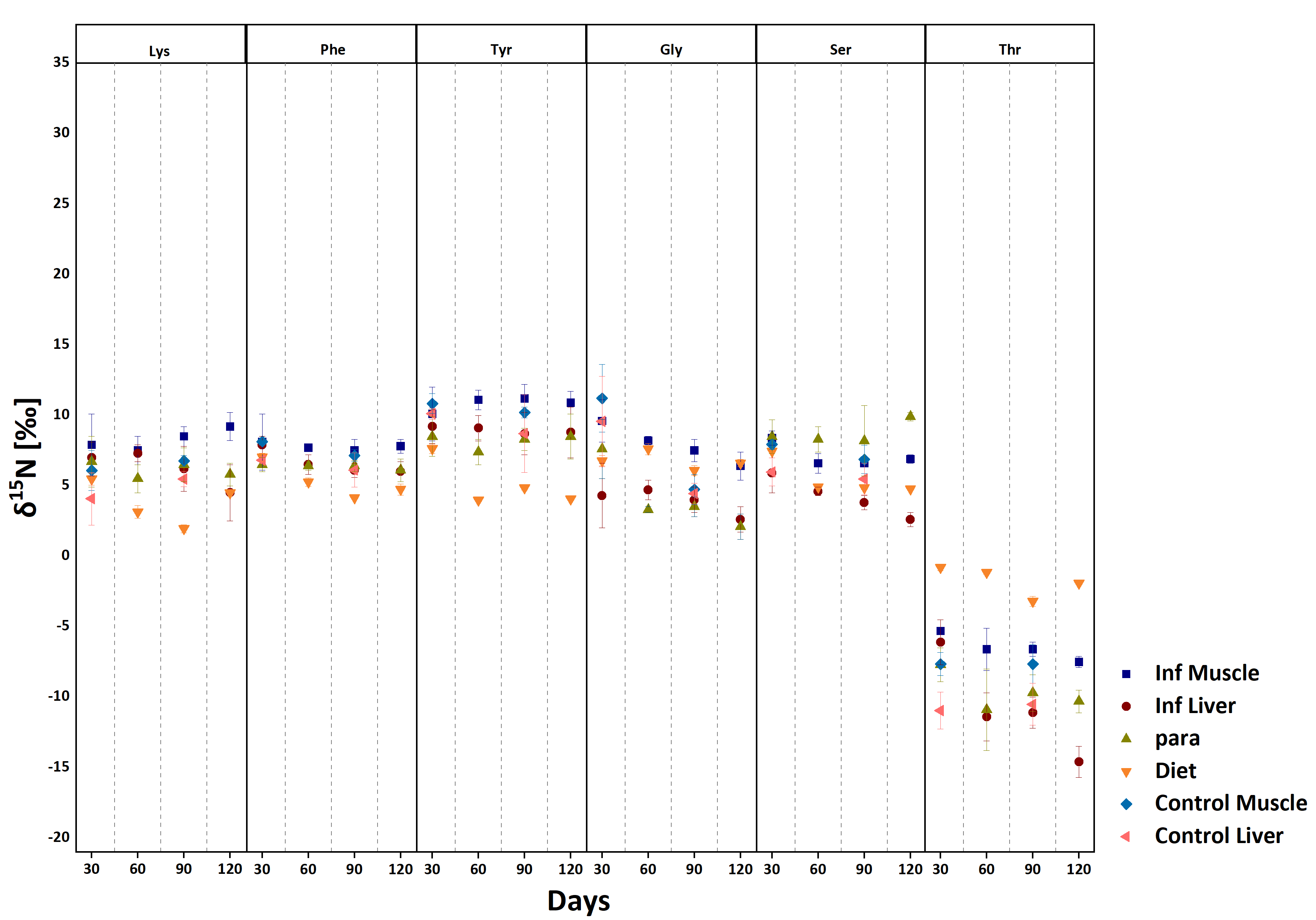
**

Table S1: Average AA δ^15^N values and standard deviations (SD) in ‰ for each sampling day of parasite (n = 4), liver (n = 4) and muscle (n = 4) samples.

| **Tissue** | **Sampling Day** | **Ala** | | **Asp** | | **Glu** | | **Ile** | | **Leu** | | **Val** | | **Met** | |
| --- | --- | --- | --- | --- | --- | --- | --- | --- | --- | --- | --- | --- | --- | --- | --- |
|  |  | Avg | SD | Avg | SD | Avg | SD | Avg | SD | Avg | SD | Avg | SD | Avg | SD |
| Parasite | 30 | 21.4 | 1.5 | 17.3 | 1.5 | 21.2 | 0.9 | 20.4 | 1.8 | 22.2 | 0.8 | 25.5 | 1.0 | - | - |
|  | 60 | 23.3 | 1.0 | 18.3 | 0.3 | 22.1 | 0.7 | 18.6 | 1.2 | 22.2 | 0.8 | 24.2 | 1.1 | - | - |
|  | 90 | 25.8 | 0.1 | 19.3 | 0.8 | 22.3 | 1.2 | 19.8 | 2.9 | 20.8 | 0.4 | 23.6 | 0.9 | - | - |
|  | 120 | 24.9 | 1.3 | 19.8 | 0.9 | 22.7 | 0.7 | 20.0 | 0.9 | 21.8 | 0.6 | 24.6 | 0.6 | - | - |
| Liver | 30 | 21.2 | 1.2 | 18.1 | 0.7 | 22.5 | 0.8 | 18.0 | 2.6 | 20.4 | 0.5 | 23.8 | 0.8 | - | - |
|  | 60 | 21.9 | 0.8 | 18.9 | 0.1 | 23.4 | 0.1 | 18.6 | 0.8 | 20.3 | 0.4 | 23.3 | 0.1 | 7.3 | 1.3 |
|  | 90 | 21.4 | 1.3 | 18.4 | 0.9 | 22.5 | 0.9 | 16.8 | 0.8 | 19.8 | 0.4 | 22.5 | 0.5 | - | - |
|  | 120 | 21.4 | 0.6 | 18.0 | 0.7 | 22.6 | 0.6 | 18.2 | 0.9 | 20.1 | 0.7 | 23.5 | 0.5 | - | - |
| Muscle | 30 | 27.7 | 0.9 | 23.2 | 1.2 | 26.9 | 0.9 | 24.3 | 1.2 | 25.8 | 0.8 | 28.2 | 0.7 | 11.6 | 1.3 |
|  | 60 | 26.8 | 0.4 | 21.5 | 0.5 | 26.3 | 0.4 | 23.7 | 0.7 | 25.6 | 0.5 | 27.3 | 0.4 | 10.9 | 1.0 |
|  | 90 | 26.1 | 0.5 | 21.9 | 0.3 | 26.2 | 0.5 | 23.0 | 1.0 | 24.8 | 0.4 | 26.8 | 0.5 | 10.4 | 1.1 |
|  | 120 | 27.0 | 0.7 | 22.1 | 0.3 | 26.9 | 0.3 | 23.3 | 0.4 | 25.0 | 0.5 | 26.9 | 0.8 | 12.1 | 1.1 |
| Diet | 30 | 16.1 | 0.3 | 14.4 | 0.4 | 16.2 | 0.5 | 13.3 | 0.1 | 11.6 | 0.0 | 16.9 | 0.2 | - | - |
|  | 60 | 16.1 | 0.1 | 12.8 | 0.3 | 17.0 | 0.3 | 12.6 | 0.2 | 9.9 | 0.1 | 16.3 | 0.3 | - | - |
|  | 90 | 14.2 | 0.1 | 10.7 | 0.4 | 14.5 | 0.2 | 10.6 | 0.1 | 9.0 | 0.5 | 14.6 | 0.3 | - | - |
|  | 120 | 15.1 | 0.3 | 11.7 | 0.2 | 15.5 | 0.4 | 11.8 | 0.3 | 9.9 | 0.3 | 15.9 | 0.1 | - | - |
| **Tissue** | **Time Point** | **Gly** | | **Lys** | | **Phe** | | **Ser** | | **Tyr** | | **Thr** | | **Pro** | |
|  |  | Avg | SD | Avg | SD | Avg | SD | Avg | SD | Avg | SD | Avg | SD | Avg | SD |
| Parasite | 30 | 7.6 | 2.1 | 6.7 | 1.8 | 6.5 | 0.5 | 8.5 | 1.2 | 8.5 | 1.4 | -7.7 | 1.2 | 15.0 | 1.2 |
|  | 60 | 3.3 | 0.2 | 5.5 | 1.0 | 6.4 | 0.3 | 8.3 | 0.9 | 7.4 | 0.9 | -10.9 | 2.9 | 16.9 | 1.4 |
|  | 90 | 3.5 | 0.7 | 6.5 | 1.2 | 6.4 | 0.4 | 8.2 | 2.5 | 8.3 | 0.8 | -9.7 | 1.3 | 11.8 | 2.5 |
|  | 120 | 2.1 | 0.9 | 5.8 | 0.8 | 6.1 | 0.8 | 9.9 | 0.3 | 8.5 | 1.6 | -10.3 | 0.8 | 13.3 | 1.8 |
| Liver | 30 | 4.3 | 2.3 | 7.0 | 1.1 | 7.9 | 0.6 | 5.9 | 1.4 | 9.2 | 1.2 | -6.1 | 1.6 | 18.4 | 2.3 |
|  | 60 | 4.7 | 0.7 | 7.3 | 0.6 | 6.5 | 0.7 | 4.6 | 0.3 | 9.1 | 0.9 | -11.4 | 1.7 | 20.3 | 1.9 |
|  | 90 | 4.0 | 0.9 | 6.2 | 1.6 | 6.1 | 0.5 | 3.8 | 0.5 | 8.7 | 1.5 | -11.1 | 1.1 | 18.3 | 2.5 |
|  | 120 | 2.6 | 0.9 | 4.5 | 2.0 | 6.0 | 0.7 | 2.6 | 0.5 | 8.8 | 1.8 | -14.6 | 1.1 | 18.3 | 1.4 |
| Muscle | 30 | 9.6 | 1.5 | 7.9 | 2.2 | 8.1 | 2.0 | 8.4 | 0.5 | 10.1 | 1.9 | -5.3 | 0.2 | 21.1 | 1.3 |
|  | 60 | 8.2 | 0.3 | 7.5 | 1.0 | 7.7 | 0.2 | 6.6 | 0.7 | 11.1 | 0.7 | -6.6 | 1.5 | 24.3 | - |
|  | 90 | 7.5 | 0.8 | 8.5 | 0.7 | 7.5 | 0.8 | 6.6 | 1.1 | 11.2 | 1.0 | -6.6 | 0.5 | 20.9 | - |
|  | 120 | 6.4 | 1.0 | 9.2 | 1.0 | 7.8 | 0.5 | 6.9 | 0.3 | 10.9 | 0.8 | -7.5 | 0.4 | 20.8 | 3.5 |
| Diet | 30 | 6.8 | 0.4 | 5.5 | 0.4 | 7.0 | 0.3 | 7.5 | 0.1 | 7.6 | 0.3 | -0.8 | 0.2 | 15.4 | 0.1 |
|  | 60 | 7.6 | 0.4 | 3.1 | 0.4 | 5.2 | 0.3 | 4.9 | 0.1 | 4.0 | 0.0 | -1.1 | 0.2 | 14.5 | 0.0 |
|  | 90 | 6.1 | 0.4 | 1.9 | 0.3 | 4.1 | 0.1 | 4.9 | 0.2 | 4.9 | 0.2 | -3.2 | 0.4 | 8.9 | 0.1 |
|  | 120 | 6.6 | 0.3 | 4.5 | 0.0 | 4.7 | 0.4 | 4.8 | 0.1 | 4.1 | 0.0 | -1.9 | 0.1 | 15.9 | 0.2 |
|  |  |  |  |  |  |  |  |  |  |  |  |  |  |  |  |

**Table S2**: Average AA δ^15^N values and standard deviations (SD) in ‰ for each sampling day of a control liver (n = 3) and muscle (n = 3) samples.

| **Tissue** | **Sampling Day** | **Ala** | | **Asp** | | **Glu** | | **Ile** | | **Leu** | | **Val** | | **Met** | |
| --- | --- | --- | --- | --- | --- | --- | --- | --- | --- | --- | --- | --- | --- | --- | --- |
|  |  | Avg | SD | Avg | SD | Avg | SD | Avg | SD | Avg | SD | Avg | SD | Avg | SD |
| Liver | 30 | 22.6 | 0.4 | 18.6 | 0.8 | 23.3 | 1.0 | 18.2 | 2.0 | 19.7 | 0.6 | 24.7 | 0.6 | - | - |
|  | 90 | 21.9 | 1.2 | 19.0 | 0.9 | 24.6 | 1.2 | 19.6 | 2.3 | 20.2 | 1.5 | 22.7 | 1.1 | - | - |
| Muscle | 30 | 29.3 | 0.8 | 23.5 | 0.2 | 27.7 | 0.2 | 24.2 | 1.0 | 26.7 | 0.8 | 27.7 | 0.5 | 9.9 | 1.4 |
|  | 90 | 26.1 | 0.8 | 22.8 | 0.4 | 27.3 | 0.4 | 23.9 | 0.6 | 25.5 | 0.7 | 27.6 | 1.2 | 11.0 | 0.5 |
| **Tissue** | **Time Point** | **Gly** | | **Lys** | | **Phe** | | **Ser** | | **Tyr** | | **Thr** | | **Pro** | |
|  |  | Avg | SD | Avg | SD | Avg | SD | Avg | SD | Avg | SD | Avg | SD | Avg | SD |
| Liver | 30 | 7.6 | 4.1 | 4.1 | 1.9 | 6.8 | 0.2 | 6.0 | 1.0 | 10.1 | 0.4 | -11.0 | 1.3 | - | - |
|  | 90 | 4.4 | 0.7 | 5.5 | 0.5 | 6.1 | 1.2 | 5.5 | 1.1 | 8.7 | 2.8 | -10.5 | 1.5 | 21.1 | 1.8 |
| Muscle | 30 | 11.2 | 2.4 | 6.1 | 1.4 | 8.1 | 0.3 | 7.9 | 0.4 | 10.8 | 0.7 | -7.7 | 0.8 | - | - |
|  | 90 | 4.7 | 1.1 | 6.8 | 0.4 | 7.1 | 0.4 | 6.9 | 1.0 | 10.2 | 0.4 | -7.7 | 1.4 | 25.1 | 4.9 |

**Table S3:** Trophic fractionation (Δδ^15^N) of AAs between parasite and liver/muscle tissue of host in ‰ for all days (N = 16) and on each sampling day (N = 4). Standard deviations are given in brackets. Significant differences of Δδ^15^N values from zero were tested individually with Pair sample t-tests (DF = 15, α = 0.05) and are marked bold. Differences for AAs in the parasite were between -1.3 to +4.6 ‰ compared to liver tissue and between -7.6 to +1.6 ‰ compared to muscle tissue.

| **AA** | **Δδ^15^N_Parasite-Liver_** | | | | | | | | | | **Δδ^15^N_Parasite-Muscle_** | | | | | | | | | |
| --- | --- | --- | --- | --- | --- | --- | --- | --- | --- | --- | --- | --- | --- | --- | --- | --- | --- | --- | --- | --- |
|  | **All Days** | | **30 Days** | | **60 Days** | | **90 Days** | | **120 Days** | | **All Days** | | **30 Days** | | **60 Days** | | **90 Days** | | **120 Days** | |
| **Ala** | ***2.1*** | ***(2.1)*** | 0.2 | (1.5) | 1.4 | (1.5) | 3.2 | (2.2) | ***3.6*** | ***(1.6)*** | ***-3.3*** | ***(2.3)*** | ***-6.3*** | ***(1.6)*** | ***-3.5*** | ***(1.1)*** | -1.4 | (1.9) | ***-2.1*** | ***(1.0)*** |
| **Asp** | ***0.3*** | ***(1.4)*** | -0.8 | (1.0) | ***-0.6*** | ***(0.4)*** | 0.9 | (1.2) | ***1.8*** | ***(0.9)*** | ***-3.3*** | ***(1.4)*** | ***-5.1*** | ***(1.0)*** | ***-3.2*** | ***(0.3)*** | ***-2.5*** | ***(0.8)*** | ***-2.4*** | ***(1.2)*** |
| **Glu** | ***-0.6*** | ***(1.0)*** | -1.2 | (0.7) | ***-1.3*** | ***(0.7)*** | -0.2 | (0.8) | 0.2 | (1.1) | ***-4.4*** | ***(1.1)*** | ***-5.7*** | ***(1.0)*** | ***-4.1*** | ***(1.1)*** | ***-3.8*** | ***(0.8)*** | ***-4.1*** | ***(0.9)*** |
| **Ile** | ***1.5*** | ***(2.3)*** | 1.2 | (3.2) | 0.0 | (0.4) | 2.9 | (2.9) | ***1.8*** | ***(1.3)*** | ***-3.9*** | ***(1.8)*** | ***-4.0*** | ***(2.2)*** | ***-5.0*** | ***(1.7)*** | -3.2 | (2.3) | ***-3.3*** | ***(0.9)*** |
| **Leu** | ***1.5*** | ***(0.6)*** | ***1.4*** | ***(0.3)*** | ***1.9*** | ***(0.7)*** | ***1.0*** | ***(0.5)*** | 1.8 | (0.3) | ***-3.6*** | ***(1.0)*** | ***-3.7*** | ***(1.4)*** | ***-3.4*** | ***(1.3)*** | ***-4.0*** | ***(0.3)*** | ***-3.1*** | ***(0.8)*** |
| **Val** | ***1.0*** | ***(1.1)*** | 1.0 | (1.9) | 0.8 | (1.1) | 1.2 | (1.0) | **1.1** | **(0.7)** | ***-2.8*** | ***(1.1)*** | ***-2.7*** | ***(1.6)*** | ***-3.1*** | ***(1.3)*** | ***-3.2*** | ***(1.0)*** | ***-2.3*** | ***(0.3)*** |
| **Pro** | ***-4.6*** | ***(2.2)*** | -5.3 | (1.7) | -3.4 | (3.2) | ***-5.0*** | ***(1.4)*** | ***-5.0*** | ***(2.4)*** | ***-7.6*** | ***(3.0)*** | ***-6.6*** | ***(1.3)*** | -8.6 | - | -10.3 | - | ***-7.4*** | ***(4.5)*** |
| **Gly** | ***-1.3*** | ***(1.4)*** | ***-2.7*** | ***(1.7)*** | ***-1.5*** | ***(0.7)*** | -0.5 | (0.7) | -0.6 | (1.1) | ***-3.7*** | ***(2.1)*** | -1.5 | (3.1) | ***-5.0*** | ***(0.3)*** | ***-3.9*** | ***(0.7)*** | ***-4.3*** | ***(1.5)*** |
| **Lys** | -0.8 | (3.3) | -1.2 | (1.0) | -3.7 | (4.4) | 0.2 | (1.8) | 1.3 | (2.8) | ***-2.7*** | ***(2.6)*** | -1.3 | (3.0) | -4.0 | (4.2) | ***-2.0*** | ***(0.8)*** | ***-3.4*** | ***(0.6)*** |
| **Phe** | -0.3 | (1.1) | ***-1.7*** | ***(0.6)*** | -0.2 | (0.7) | 0.4 | (0.9) | 0.1 | (1.2) | ***-1.4*** | ***(1.1)*** | -1.6 | (1.6) | ***-1.3*** | ***(0.3)*** | -1.0 | (1.2) | -1.7 | (1.2) |
| **Ser** | ***4.4*** | ***(2.4)*** | 2.2 | (1.9) | ***3.7*** | ***(1.2)*** | ***4.3*** | ***(2.0)*** | ***7.3*** | ***(0.8)*** | ***1.6*** | ***(1.9)*** | 0.1 | (0.9) | 1.7 | (1.6) | 1.6 | (2.9) | ***3.0*** | ***(0.5)*** |
| **Tyr** | -0.8 | (1.7) | -0.9 | (2.5) | ***-1.7*** | ***(0.7)*** | -0.4 | (2.0) | -0.3 | (1.5) | ***-2.6*** | ***(1.3)*** | -1.6 | (1.5) | ***-3.7*** | ***(0.3)*** | ***-2.9*** | ***(0.4)*** | -2.3 | (1.5) |
| **Thr** | 1.2 | (2.9) | -1.8 | (1.6) | 1.0 | (3.7) | 1.4 | (1.2) | ***4.3*** | ***(1.3)*** | ***-2.8*** | ***(1.4)*** | ***-2.4*** | ***(1.3)*** | -2.8 | (2.3) | ***-3.1*** | ***(1.6)*** | ***-2.8*** | ***(0.8)*** |

**Table S4**: Trophic fractionation (Δδ^15^N in ‰) of AAs between infected host tissues (n = 4) and control host tissues (n=3) for 30 and 90 days. Standard errors of the mean difference are given in brackets. Host tissues = muscle and liver tissues

| **AA** | **Δδ^15^N_Inf Muscle- Control Muscle_** | | | | | **Δδ^15^N_Inf Liver- Control Liver_** | | | | |
| --- | --- | --- | --- | --- | --- | --- | --- | --- | --- | --- |
|  | **30 Days 90 Days** | | | | | **30 Days 90 Days** | | | | |
| **Ala** | -1.6 | (0.7) | | 0.0 | (0.5) | -1.4 | (0.6) | -0.5 | (0.9) |  |
| **Asp** | -1.1 | (0.6) | | -1.0 | (0.3) | -0.5 | (0.6) | -0.6 | (0.7) |  |
| **Glu** | -0.8 | (0.5) | | -1.2 | (0.4) | -0.8 | (0.7) | -2.2 | (0.9) |  |
| **Ile** | 0.1 | (0.8) | | -0.9 | (0.6) | -0.2 | (1.8) | -2.8 | (1.4) |  |
| **Leu** | -0.8 | (0.6) | | -0.6 | (0.4) | 0.7 | (0.4) | -0.4 | (0.9) |  |
| **Val** | 0.5 | (0.5) | -0.8 | | (0.8) | -1.0 | (0.5) | -0.2 | (0.7) |  |
| **Pro** | - |  | -4.2 | | (2.9) | -4.6 | (1.3) | -2.8 | (1.6) |  |
| **Gly** | -2.1 | (1.6) | 2.7 | | (0.7) | -5.2 | (2.2) | -0.4 | (0.6) |  |
| **Lys** | 1.9 | (1.4) | 1.7 | | (0.4) | 2.9 | (2.1) | 0.8 | (0.8) |  |
| **Phe** | 0.0 | (1.0) | 0.4 | | (0.5) | 1.1 | (0.3) | 0.0 | (0.7) |  |
| **Ser** | 0.5 | (0.3) | -0.3 | | (0.8) | -0.1 | (0.9) | -1.6 | (0.7) |  |
| **Tyr** | -0.7 | (1.0) | 0.9 | | (0.5) | -0.9 | (0.7) | -0.1 | (1.8) |  |
| **Thr** | 2.3 | (0.5) | 1.1 | | (0.8) | 4.8 | (1.1) | -0.6 | (1.0) |  |

**Table S5**: Trophic fractionation (Δδ^15^N in ‰) of AAs between control host tissues (n=3) and diet (n=1) for 30 and 90 days. Standard errors of the mean difference are given in brackets. Host tissues = muscle and liver tissues

| **AA** | **Δδ^15^N_Control Muscle-Diet_** | | | | **Δδ^15^N_Control Liver-Diet_** | | | | |
| --- | --- | --- | --- | --- | --- | --- | --- | --- | --- |
|  | **30 Days 90 Days** | | | | **30 Days 90 Days** | | | | |
| **Ala** | 13.1 | (0.6) | 11.9 | (0.5) | 6.4 | (0.4) | 7.8 | (0.7) |  |
| **Asp** | 9.1 | (0.4) | 12.1 | (0.5) | 4.3 | (0.6) | 8.3 | (0.7) |  |
| **Glu** | 11.5 | (0.5) | 12.8 | (0.5) | 7.2 | (0.8) | 10.1 | (0.8) |  |
| **Ile** | 11.0 | (0.6) | 13.3 | (0.4) | 4.9 | (1.2) | 9.0 | (1.3) |  |
| **Leu** | 15.1 | (0.5) | 16.5 | (0.6) | 8.1 | (0.3) | 11.2 | (1.0) |  |
| **Val** | 10.8 | (0.4) | 13.0 | (0.8) | 7.8 | (0.4) | 8.0 | (0.7) |  |
| **Pro** | - |  | 16.1 | (2.8) | 7.6 | (0.1) | 12.2 | (1.0) |  |
| **Gly** | 4.4 | (1.4) | -1.3 | (0.7) | 2.8 | (1.9) | -1.6 | (0.6) |  |
| **Lys** | 0.6 | (0.9) | 4.8 | (0.4) | -1.4 | (1.2) | 3.5 | (0.4) |  |
| **Phe** | 1.1 | (0.3) | 3.0 | (0.3) | -0.2 | (0.3) | 2.0 | (0.7) |  |
| **Ser** | 0.5 | (0.2) | 2.0 | (0.6) | -1.5 | (0.6) | 0.6 | (0.7) |  |
| **Tyr** | 3.2 | (0.5) | 5.3 | (0.3) | 2.5 | (0.4) | 3.9 | (1.6) |  |
| **Thr** | -6.9 | (0.5) | -4.5 | (0.9) | -10.2 | (0.8) | -7.3 | (0.9) |  |

**Table S6**: Trophic fractionation (Δδ^15^N in ‰) of AAs between infected host tissues (n=4) and diet (n=1) for 30, 60, 90, and 120 days. Standard errors of the mean difference are given in brackets. Host tissues = muscle and liver tissues

| **AA** | **Δδ^15^N**_In_**_fected Muscle - Diet_** | | | | | | | | **Δδ^15^N_Infected Liver - Diet_** | | | | **Δδ^15^N**_Parasite -_ **_Diet_** | | | | | | | |  |
| --- | --- | --- | --- | --- | --- | --- | --- | --- | --- | --- | --- | --- | --- | --- | --- | --- | --- | --- | --- | --- | --- |
|  | **30 Days** | | **60 Days** | | **90 Days** | | **120 Days** | | **30 Days** | **60 Days** | **90 Days** | **120 Days** | **60 Days** | | **90 Days** | | **120 Days** | | **120 Days** | | |
| **Ala** | 11.5 | (0.5) | 10.7 | (0.2) | 11.9 | (0.3) | 11.9 | (0.5) | 5.0 (0.7) 5.8 (0.4) 7.3 (0.7) 6.2 (0.4) | | | | 5.2 | (0.8) | 7.2 | (0.5) | 11.6 | (0.1) | 9.8 | (0.7) | |
| **Asp** | 8.0 | (0.7) | 8.7 | (0.4) | 11.2 | (0.5) | 10.4 | (0.2) | 3.7 (0.5) 6.1 (0.3) 7.7 (0.6) 6.3 (0.4) | | | | 2.9 | (0.9) | 5.5 | (0.3) | 8.7 | (0.6) | 8.1 | (0.5) | |
| **Glu** | 10.7 | (0.7) | 9.3 | (0.3) | 11.6 | (0.3) | 11.3 | (0.4) | 6.3 (0.6) 6.4 (0.3) 8.0 (0.5) 7.0 (0.5) | | | | 5.1 | (0.7) | 5.1 | (0.5) | 7.8 | (0.7) | 7.2 | (0.5) | |
| **Ile** | 11.1 | (0.6) | 11.1 | (0.4) | 12.4 | (0.5) | 11.5 | (0.3) | 4.7 (1.3) 6.0 (0.5) 6.2 (0.4) 6.4 (0.5) | | | | 7.1 | (0.9) | 6.0 | (0.7) | 9.2 | (1.5) | 8.2 | (0.5) | |
| **Leu** | 14.2 | (0.4) | 15.7 | (0.3) | 15.8 | (0.5) | 15.1 | (0.4) | 8.8 (0.2) 10.4 (0.2) 10.7 (0.5) 10.2 (0.4) | | | | 10.6 | (0.4) | 12.3 | (0.4) | 11.8 | (0.5) | 11.9 | (0.4) | |
| **Val** | 11.3 | (0.4) | 11.0 | (0.4) | 12.1 | (0.4) | 11.1 | (0.4) | 6.9 (0.5) 7.0 (0.3) 7.8 (0.4) 7.7 (0.3) | | | | 8.6 | (0.5) | 7.8 | (0.6) | 9.0 | (0.6) | 8.8 | (0.3) | |
| **Pro** | 5.7 | (0.7) | - |  | 11.9 | (0.1) | 5.0 | (1.7) | 3.0 (1.1) 5.8 (0.9) 9.3 (1.2) 2.4 (0.7) | | | | 2.5 | (3.0) | 2.4 | (0.7) | 2.9 | (1.2) | -2.6 | (0.9) | |
| **Gly** | 2.3 | (0.9) | 0.6 | (0.7) | 1.4 | (0.6) | -0.2 | (0.6) | -1.3 (0.4) -2.9 (0.5) -2.0 (0.6) -4.0 (0.5) | | | | 0.0 | (0.9) | -4.3 | (0.4) | -2.6 | (0.5) | -4.5 | (0.5) | |
| **Lys** | 2.5 | (1.2) | 4.4 | (0.7) | 6.5 | (0.4) | 4.7 | (0.5) | 1.5 (0.7) 4.1 (0.8) 4.3 (0.5) 0.0 (1.0) | | | | 1.2 | (1.0) | 2.4 | (0.7) | 4.5 | (0.6) | 1.3 | (0.4) | |
| **Phe** | 1.1 | (1.0) | 2.4 | (0.3) | 3.3 | (0.4) | 3.1 | (0.5) | 0.9 (0.4) 1.3 (0.4) 2.0 (0.3) 1.2 (0.5) | | | | -0.5 | (0.4) | 1.1 | (0.3) | 2.3 | (0.2) | 1.3 | (0.6) | |
| **Ser** | 0.9 | (0.3) | 1.6 | (0.4) | 1.7 | (0.6) | 2.1 | (0.1) | -1.6 (0.7) -0.3 (0.2) -1.0 (0.3) -2.2 (0.2) | | | | 1.0 | (0.6) | 3.4 | (0.5) | 3.3 | (1.2) | 5.1 | (0.2) | |
| **Tyr** | 2.5 | (1.0) | 7.0 | (0.4) | 6.3 | (0.5) | 6.8 | (0.4) | 1.6 (0.7) 5.1 (0.4) 3.8 (0.8) 4.8 (0.9) | | | | 0.9 | (0.8) | 3.4 | (0.5) | 3.4 | (0.4) | 4.4 | (0.8) | |
| **Thr** | -4.5 | (0.3) | -5.4 | (0.7) | -3.4 | (0.4) | -5.6 | (0.2) | -5.3 (0.8) -10.3 (0.8) -7.9 (0.7) -12.7 (0.6) | | | | --6.9 | (0.6) | -9.7 | (1.5) | -6.5 | (0.7) | -8.4 | (0.4) | |

1 Yarnes, C. T. & Herszage, J. The relative influence of derivatization and normalization procedures on the compound-specific stable isotope analysis of nitrogen in amino acids. *Rapid Commun Mass Spectrom* **31**, 693-704, doi:10.1002/rcm.7832 (2017).

2 Kim, D. *et al.* Environmental fate and trophic transfer of synthetic musk compounds and siloxanes in Geum River, Korea: Compound-specific nitrogen isotope analysis of amino acids for accurate trophic position estimation. *Environment International* **161**, doi:10.1016/j.envint.2022.107123 (2022).

3 Ishikawa, N. F. *et al.* Stable nitrogen isotopic composition of amino acids reveals food web structure in stream ecosystems. *Oecologia* **175**, 911-922, doi:10.1007/s00442-014-2936-4 (2014).

4 Zhang, Z. *et al.* A reliable compound-specific nitrogen isotope analysis of amino acids by GC-C-IRMS following derivatisation into N-pivaloyl-iso-propyl (NPIP) esters for high-resolution food webs estimation. *J Chromatogr B Analyt Technol Biomed Life Sci* **1033-1034**, 382-389, doi:10.1016/j.jchromb.2016.09.004 (2016).

5 Riekenberg, P. M., van der Meer, M. & Schouten, S. Practical considerations for improved reliability and precision during determination of δ^15^N values in amino acids using a single combined oxidation-reduction reactor. *Rapid Commun Mass Spectrom* **34**, e8797, doi:10.1002/rcm.8797 (2020).
